# A Mitochondrial Polymorphism Alters Immune Cell Metabolism and Protects Mice from Skin Inflammation

**DOI:** 10.3390/ijms22031006

**Published:** 2021-01-20

**Authors:** Paul Schilf, Axel Künstner, Michael Olbrich, Silvio Waschina, Beate Fuchs, Christina E. Galuska, Anne Braun, Kerstin Neuschütz, Malte Seutter, Katja Bieber, Lars Hellberg, Christian Sina, Tamás Laskay, Jan Rupp, Ralf J. Ludwig, Detlef Zillikens, Hauke Busch, Christian D. Sadik, Misa Hirose, Saleh M. Ibrahim

**Affiliations:** 1 Luebeck Institute of Experimental Dermatology, University of Luebeck, 23562 Luebeck, Germany; Paul.Schilf@uksh.de (P.S.); axel.kuenstner@uni-luebeck.de (A.K.); michael.olbrich@uni-luebeck.de (M.O.); kerstinneuschutz@hotmail.com (K.N.); Katja.Bieber@uksh.de (K.B.); Ralf.Ludwig@uksh.de (R.J.L.); hauke.busch@uni-luebeck.de (H.B.); 2 Institute of Cardiogenetics, University of Luebeck, 23562 Luebeck, Germany; 3 Institute of Human Nutrition and Food Science, Christian-Albrechts-University of Kiel, 24098 Kiel, Germany; s.waschina@nutrinf.uni-kiel.de; 4 Leibniz-Institute for Farm Animal Biology (FBN), Core Facility Metabolomics, 18196 Dummerstorf, Germany; fuchs.beate@fbn-dummerstorf.de (B.F.); galuska.christina@fbn-dummerstorf.de (C.E.G.); 5 Department of Dermatology, University of Luebeck, 23562 Luebeck, Germany; annebraun@fami-braun.de (A.B.); m.seutter@outlook.de (M.S.); Detlef.Zillikens@uksh.de (D.Z.); Christian.Sadik@uksh.de (C.D.S.); 6 Department of Infectious Diseases and Microbiology, University of Luebeck, 23562 Luebeck, Germany; hellberg@gmx.de (L.H.); Tamas.Laskay@uksh.de (T.L.); Jan.Rupp@uksh.de (J.R.); 7 Institute of Nutritional Medicine, University of Luebeck, 23562 Luebeck, Germany; Christian.Sina@uksh.de; 8 Center for Research on Inflammation of the Skin (CRIS), University of Luebeck, 23562 Luebeck, Germany; 9 College of Medicine and Sharjah Institute for Medical Research, University of Sharjah, 27272 Sharjah, UAE

**Keywords:** conplastic mice, mtDNA polymorphisms, mitochondria, immunometabolism, metabolomics, autoimmune disease, skin inflammation, Complex V, ATP8, *mt-Atp8*, short chain fatty acids, propionate

## Abstract

Several genetic variants in the mitochondrial genome (mtDNA), including ancient polymorphisms, are associated with chronic inflammatory conditions, but investigating the functional consequences of such mtDNA polymorphisms in humans is challenging due to the influence of many other polymorphisms in both mtDNA and the nuclear genome (nDNA). Here, using the conplastic mouse strain B6-mt^FVB^, we show that in mice, a maternally inherited natural mutation (m.7778G > T) in the mitochondrially encoded gene ATP synthase 8 (*mt-Atp8*) of complex V impacts on the cellular metabolic profile and effector functions of CD4^+^ T cells and induces mild changes in oxidative phosphorylation (OXPHOS) complex activities. These changes culminated in significantly lower disease susceptibility in two models of inflammatory skin disease. Our findings provide experimental evidence that a natural variation in mtDNA influences chronic inflammatory conditions through alterations in cellular metabolism and the systemic metabolic profile without causing major dysfunction in the OXPHOS system.

## 1. Introduction

Mitochondrial gene variations have been reported to be associated not only with classical mitochondrial diseases, such as Leber’s hereditary optic neuritis (LHON) [1], but also with common diseases in humans, as well as model organisms. These include ageing and age-related diseases, neurodegenerative diseases, metabolic diseases, and chronic inflammation [2].

Direct links between specific sets of mitochondrial genome (mtDNA) variations, i.e., mitochondrial haplogroups, and functional consequences have been reported in mitochondrial disorders so-called classic mitochondrial diseases [3]. The functional consequences of single mtDNA variations have been reported in mice carrying a single mtDNA mutation in the NADH dehydrogenase 6 gene (*mt-Nd6*) gene, which are used as murine model of LHON [4].

The roles of ancient mtDNA polymorphisms in longevity and aging phenotypes, which are common disease phenotypes, in mouse models were recently described by us and others [5,6,7,8,9,10]. While the functional impact of such natural mtDNA variations on inflammatory conditions and more specifically on immune cells requires more detailed investigation, the involvement of nuclear-encoded mitochondrial genes in immune cell functions has been confirmed. For example, the gene *Cox5b*, which encodes cytochrome c oxidase subunit 5B protein of mitochondrial complex IV, plays a role in the function of a murine macrophage cell line [11] and *Uqcrfs1*, which encodes the Rieske iron-sulfur protein (RISP) protein, an essential subunit of mitochondrial complex III, and is involved in the function of murine CD4^+^ T cells [12] and murine haematopoietic stem cells [13].

The relevance of mtDNA variants to immune cell responses was previously investigated. In 1990, a study demonstrated that amino acid substitutions at positions 2766 and 2767 at the 5′ end of the NADH dehydrogenase 1 gene are recognized by cytotoxic T cells in mice [14]. Later, mouse transplantation experiments demonstrated that tumour cell cybrids and non-tumour embryonic stem cell cybrids carrying only allogeneic mtDNA are rejected and cleared out by natural killer T (NKT) cells in the recipient mouse [15]. This study compared mtDNA from NZB/NSlc mice carrying multiple mtDNA variants with that from B6 mice. These studies suggest the antigenic potential of mitochondrial proteins/peptides derived from mtDNA variants.

In parallel, immunometabolism, a field of immunology that focuses on cellular metabolism (i.e., biochemical processes) in immune cells, has emerged in recent years. The intricate regulation of the immune system is underlined by diverse cellular metabolic demands. Metabolic activity is tuned to fit the demands of immune cells and their functions. Conversely, the changes in small molecule/metabolite abundance and metabolic function affect the function of immune cells. Cellular metabolism plays an important role in sustaining their energy demand of immune cells. In recent years, the diverse spectrum of metabolic pathway requirements for different immune cell subsets has become an important research topic related to immunological function [16,17]. Activation and differentiation both require and result in distinct metabolic states in different immune cell subsets, such as CD8^+^ T cell subsets [18,19,20,21], effector and regulatory CD4^+^ T cell subsets [22,23,24,25], and macrophages [26,27]. As such, there is accumulating evidence for a close interaction and a mutual dependence between cellular metabolism pathways and immune responses. Mitochondria, which are the most potent biological generators of energy and act as metabolic intermediates, are directly and/or indirectly involved in shaping immune cell phenotypes and functions; consequently, immune responses lead to immune-related pathological conditions, including inflammation [28,29]. However, the effects of mtDNA variations and their functional consequences on immune responses and inflammation are largely elusive, partly due to the parallel impact of the highly polymorphic nuclear genome.

Conplastic mouse strains, mouse lines that carry distinct mtDNA variants derived from different common inbred mouse strains on the same nuclear genome background, eliminating the parallel influence of the highly polymorphic nuclear genome, are powerful tools for investigating the functional consequences of mtDNA variations. The mitochondrial genomes of common inbred mouse strains exhibit unique variations between strains, but stem from a common ancestral female mouse [30,31,32,33]. We previously generated a B6-mt^FVB^ conplastic mouse strain, which carries a stable and functionally relevant single nucleotide variant in the mitochondrially encoded gene ATP8 synthase 8 (*mt-Atp8,* m.7778G > T; D5Y) not carried by C57BL/6J (B6-mt^B6^; B6) mice [33] (Table S1). This gene encodes the subunit A6L of the ATP synthase (complex V) of the oxidative phosphorylation (OXPHOS) system.

Here, using B6-mt^FVB^ mice, we revealed that the m.7778G > T gene variant led to subtle changes in mitochondrial OXPHOS functions accompanied by alterations in the systemic metabolic profile and cellular metabolism in immune cells, specifically CD4^+^ T cells. These changes resulted in the modulation of the effector function of CD4^+^ T cells and differential disease susceptibility in experimental inflammatory skin diseases in the mice, namely antibody-transfer autoimmune skin blistering disease epidermolysis bullosa acquisita (EBA) and imiquimod (IMQ)-induced psoriasiform dermatitis. These skin disease models were selected because these are well-established prototypical mouse models of skin inflammatory conditions [34,35]. These findings provide the first evidence that adaptive polymorphisms in mitochondrial genes that cause minimal functional changes in the OXPHOS machinery can significantly modulate systemic and cellular metabolism in immune cells, thus contributing to the emergence of complex chronic inflammatory diseases.

## 2. Results

### 2.1. The Natural Polymorphism m.7778G > T in the mt-Atp8 Gene Influences Mitochondrial OXPHOS Function to a Moderate Extent

**Figure 1:**
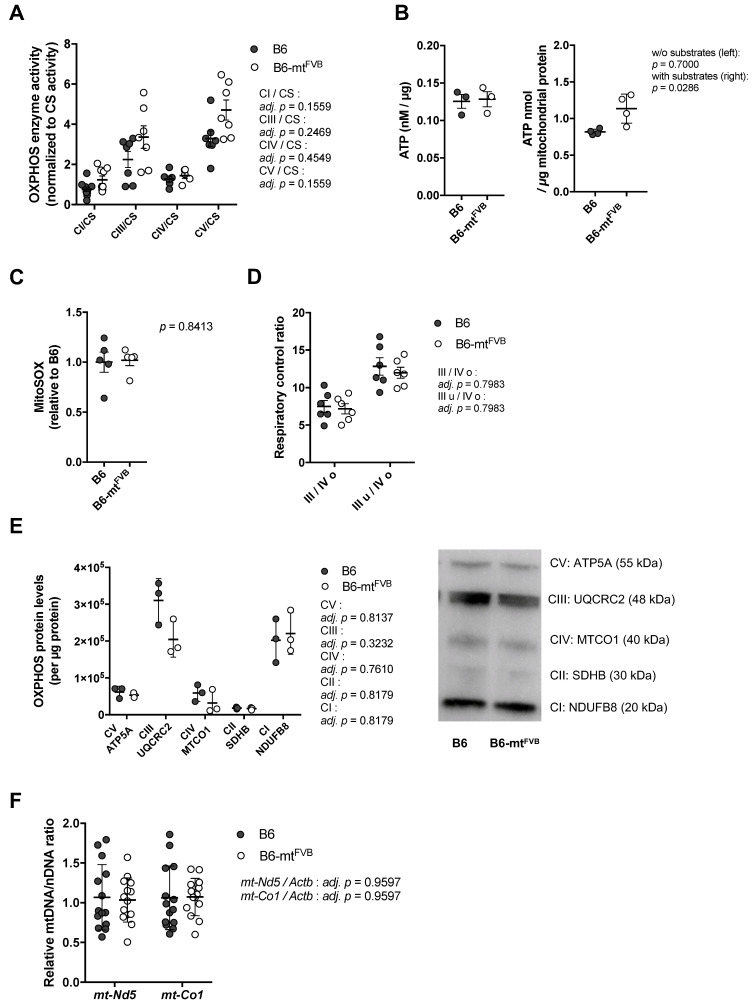
A natural polymorphism in the mitochondrially encoded ATP synthase 8 (*mt-Atp8*) gene (m.7778G > T) has minor impact on mitochondrial oxidative phosphorylation (OXPHOS) function. (**A**) Levels of oxidative phosphorylation (OXPHOS) complex enzyme activities in liver mitochondria obtained from B6-mt^FVB^ and B6 mice (age, sex) were normalized to the individual citrate synthase (CS) level. Complex I, III and V activities normalized to the CS (CI/CS, CIII/CS, and CV/CS, respectively) in liver mitochondria from B6-mt^FVB^ mice showed a trend of higher levels compared with those from B6 mice. Adj. *p* = 0.1559 (CI/CS), adj. *p* = 0.2469 (CIII/CS), adj. *p* = 0.4549 (CIV/CS), and adj. *p* = 0.1559 (CV/CS), multiple *t* test. (**B**) ATP production in liver mitochondria, under supplementation with substrates. Liver mitochondria were incubated for 30 min with (right) or without (left) substrates before addition of luciferase reaction buffer. The values of chemiluminescence were detected. Liver mitochondria from B6-mt^FVB^ mice showed higher ATP levels compared with those from B6 mice. Without substrate, *p* = 0.7000; with substrates, *p* = 0.0286, Mann–Whitney *U* test. Females, 3 months old, *n* = 3–4/strain. (**C**) Mitochondrial superoxide in liver mitochondria was determined using MitoSOX^TM^ with supplementation of substrates. Liver mitochondria were incubated with the substrates for 30 min before the reaction of signal intensity. Liver mitochondria from B6-mt^FVB^ mice exhibited comparable levels of mitochondrial superoxide compared with those from B6 mice. *p* = 0.8413, Mann–Whitney *U* test. (**D**) Oxygen consumption levels were determined in liver mitochondria using Seahorse XF analyzer, and respiratory control ratios were calculated. No differences were observed between the strains. III/VI o; a ratio of state III (oxygen consumption rate under ADP supplementation) to state IV o (oxygen consumption rate under oligomycin supplementation), III u/IV o; a ratio of state III u (oxygen consumption rate under uncoupler FCCP supplementation) to state IV o. Adj. *p* = 0.7983, respectively, multiple *t* test. (**E**) Right: Quantified values of Western blotting of liver mitochondria samples display no significant difference in mitochondrial OXPHOS subunits protein levels between the strains. Adj. *p* = 0.8137 (CV, complex V), adj. *p* = 0.3232 (CIII, complex III), adj. *p* = 0.7610 (CIV, complex IV), adj. *p* = 0.8179 (CII, complex II), and adj. *p* = 0.8179 (CI, complex I); multiple *t* test. Left: a representative blot picture. Two months old, females, *n* = 3/strain. (**F**) The relative mtDNA levels to nDNA levels in liver DNA samples obtained from B6-mt^FVB^ and B6 mice were determined using qPCR by amplification of *mt-Nd5* and *mt-Co1* genes, and the beta-actin gene (*Actb*). Each mtDNA gene level was normalized to the level of the *Actb*. No significant difference was observed between the strains. Adj. *p* = 0.9597 (*mt-Nd5/Actb* and *mt-Co1/Actb*), multiple *t* test. *N* = 14/strain (7 males and 7 females; 3 to 4 months of age).

To assess the impact of the natural polymorphism m.7778G > T in the *mt-Atp8* gene on mitochondrial function, liver mitochondria were isolated from B6-mt^FVB^ and B6 mice. The activities of oxidative phosphorylation (OXPHOS) complexes (complex I; CI, complex III; CIII, complex IV; CIV, and complex V; CV) and the enzymatic activity of citrate synthase (CS) activities were measured. Liver mitochondria prepared from B6-mt^FVB^ mice demonstrated a trend towards higher levels of CI, CIII, and CV activities normalized to CS level than those isolated from B6 mice (Figure 1A). To evaluate whether the observed slight increases in OXPHOS complex activities in B6-mt^FVB^ mitochondria are associated with ATP production, we measured ATP levels in liver mitochondria in both a tricarboxylic acid (TCA) substrate-rich environment (i.e., supplementation of ADP, pyruvate, malate, and glutamate) and minimal substrate assay buffer. While the ATP levels measured in the standard buffer were comparable between the two strains, those detected in substrate-rich buffer were significantly higher for liver mitochondria isolated from B6-mt^FVB^ mice than for those isolated from B6 mice (Figure 1B). The level of mitochondrial superoxide, a by-product of the respiratory chain, was also measured in liver mitochondria, and the levels were comparable between liver mitochondria from the B6-mt^FVB^ mice and those from B6 mice (Figure 1C). At the same time, hydrogen peroxide levels in the same liver mitochondria samples were measured and were found to be similar between the strains (Figure S1A). We also observed relatively higher expression of Superoxide dismutase 2 (*Sod2*) gene in liver mitochondria prepared from B6-mt^FVB^ mice than those isolated from B6 mice (Figure S1B). The respiratory control ratio, which was determined by the oxygen consumption rate (OCR), was comparable between liver mitochondria from B6-mt^FVB^ mice and those from B6 mice (Figure 1D). Protein levels of OXPHOS complex subunits in liver mitochondria were also comparable between the strains (Figure 1E). In line with these unaltered OXPHOS protein levels, the ratio of the relative mtDNA copy number to the nDNA copy number in genomic liver DNA was also unchanged between the strains (Figure 1F).

These findings in isolated mitochondria suggest that the natural single nucleotide variant m.7778G > T in the *mt-Atp8* gene has a very mild impact on mitochondrial OXPHOS function. In liver mitochondria carrying the mtDNA variant, slightly increased OXPHOS activity leaves mitochondrial function unaffected, unless exposed to a supra-physiological substrate.

### 2.2. The Natural Variant m.7778G > T Results in a Differential Cellular Respiration Profile that Consequently Modified Cytokine Production in CD4^+^ T Cells

**Figure 2:**
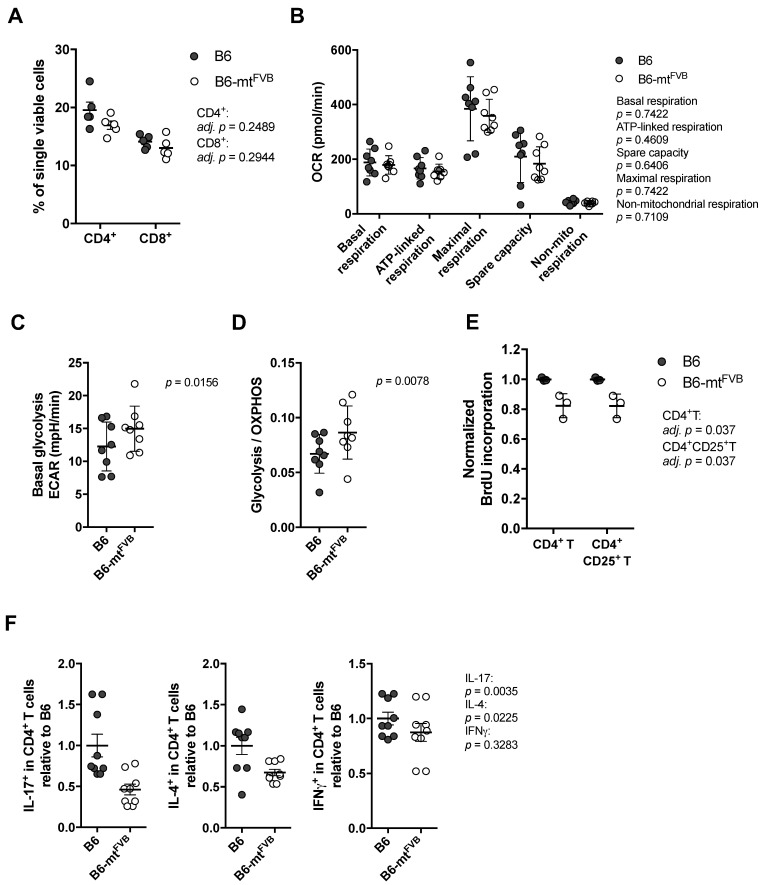
The natural variation m.7778G > T in the *mt-Atp8* gene alters the respiratory profile in CD4^+^ T cells. (**A**) The proportion of CD4^+^ T cells and CD8^+^ T cells in the spleen from B6-mt^FVB^ was comparable to that from B6 mice. CD4^+^ T cells, adj. *p* = 0.2489; CD8^+^ T cells, adj. *p* = 0.2944; multiple *t* test. *N* = 5 (2 males and 3 females)/strain, 2 to 3 months old. (**B**) Oxygen consumption rate (OCR) in CD4^+^ T cells from B6-mt^FVB^ and B6 mice were determined using the Seahorse XF bioanalyzer. Levels of calculated basal respiration, ATP-linked respiration, maximal respiration, and spare capacity, as well as non-mitochondrial respiration, were comparable between the cells from B6-mt^FVB^ and those from B6 mice. *p* = 0.7422 (basal respiration), *p* = 0.4609 (ATP-linked respiration), *p* = 0.6406 (spare capacity), *p* = 0.7422 (maximal respiration), *p* = 0.7109 (non-mitochondrial respiration), Wilcoxon matched-pairs test. *N* = 8 (7 males and 1 female)/strain, sex-matched assay in each test. Three to 4 months of age. (**C**) Extra cellular acidification rate (ECAR) was determined in the same experiments of (B). Basal glycolysis levels were calculated, and the levels in CD4^+^ T cells isolated from B6-mt^FVB^ were significantly higher than those in cells from B6 mice. *p* = 0.0156, Wilcoxon matched-pairs test. (**D**) A ratio of basal glycolysis levels to basal respiration levels in CD4^+^ T cells was calculated, and that of B6-mt^FVB^ mice exhibited significantly higher ratio than that of B6. Values were obtained from the same experiments of (A). *p* = 0.0078, Wilcoxon matched-pairs test. (**E**) Cell proliferation upon the activation with anti-mouse CD3 and anti-mouse CD28 antibodies was determined in CD4^+^ T cells isolated from B6-mt^FVB^ and B6 mice. Incorporated bromodeoxyuridine (BrdU) levels normalized to the average of those in cells from B6 mice are displayed. BrdU levels in CD4^+^ T cells and CD4^+^ CD25^+^ T cells from B6-mt^FVB^ mice were significantly lower than those from B6 mice. Adj. *p* = 0.037, respectively. Multiple *t* test. *N* = 3/strain, males, 3 months of age. (**F**) Intracellular cytokine levels were determined in activated splenocytes using anti-CD3 and anti-CD28 antibodies. Cell proportions (% in CD4^+^ T cells) normalized to average of those in B6 are displayed. The proportion of IL-17^+^ CD4^+^ T cells and IL-4^+^ CD4^+^ T cells were significantly lower in B6-mt^FVB^ mice compared to B6 mice (*p* = 0.0035 and *p* = 0.0225, respectively), while that of interferon gamma (IFNγ)^+^ CD4^+^ T cells was comparable between the strains (*p* = 0.3283). Mann–Whitney *U* test, *n* = 9/strain, male, 3 months old.

Next, we investigated the impact of the m.7778G > T polymorphism at the cellular level. For this purpose, we evaluated CD4^+^ T cells, which are indispensable in a number of autoimmune diseases. First, we evaluated the proportion of T cell subpopulations in the spleens of B6-mt^FVB^ and B6 mice. Both strains showed similar proportion of CD4^+^ T and CD8^+^ T cells (Figure 2A). Splenic CD4^+^ T cells were prepared, and their cellular metabolism was evaluated by flux analysis using a Seahorse XF analyser. The levels of basal respiration, ATP-linked respiration, maximal respiration, spare capacity, and non-mitochondrial respiration levels calculated by the OCR were comparable between CD4^+^ T cells from the two strains (Figure 2B). However, basal glycolysis levels in CD4^+^ T cells from B6-mt^FVB^ mice were significantly higher than those in cells from B6 mice (Figure 2C), and the ratio of basal glycolysis to basal OXPHOS respiration was higher in cells from B6-mt^FVB^ mice than cells from B6 mice (Figure 2D). An alteration in the cellular respiratory profile, i.e., increased glycolysis levels compared to OXPHOS respiration, similar to that found in CD4^+^ T cells from B6-mt^FVB^ mice was observed in B cells from B6-mt^FVB^ mice (Figure S2A,B). ATP levels in CD4^+^ T cells before and after activation, which were determined in a standard assay medium, were comparable between the strains, and no significant difference was detected between cells from B6-mt^FVB^ and those from B6 mice at either point (Figure S2C). The levels of mitochondrial superoxide and the mitochondrial membrane potential in activated T cells were unaltered between the two strains (Figure S2D,E). To elucidate the functional consequences of the altered cellular respiratory profile in CD4^+^ T cells in B6-mt^FVB^ mice, cellular proliferation levels in peripheral lymphocytes after T cell activation with anti-mouse CD3 and anti-mouse CD28 antibodies were evaluated. The activated CD4^+^ T cell (CD4^+^ CD25^+^ T cell) population, as well as the total CD4^+^ T cell population, in B6-mt^FVB^ mice exhibited significantly less cell proliferation than the same cell populations in B6 mice (Figure 2E), while the total cell proportion was unchanged between the strains (Figure S2F). This phenomenon seen in CD4^+^ T cells was not observed in CD8^+^ T cell populations (Figure S2G). We further evaluated cytokine levels in CD4^+^ T cells. Intracellular staining of interleukin (IL)-17, IL-4, and interferon gamma (IFN*γ*) in the CD4^+^ T cell population revealed that the proportions of IL-17^+^ cells and IL-4^+^ in B6-mt^FVB^ mice were significantly smaller than those in B6 mice (Figure 2F), while there was no difference in IFN*γ*^+^ CD4^+^ T cell populations between strains (Figure 2F). The proportion of regulatory T cells in B6-mt^FVB^ mice was comparable to that in B6 mice (Figure S2H). Additionally, the impact of the *mt-Atp8* variant on neutrophil granulocytes (neutrophils) was assessed because this immune cell population plays a critical role in the effector phases of many inflammatory conditions, including skin inflammation. No significant difference in neutrophil function was observed between B6-mt^FVB^ and B6 mice (Figure S2I).

These findings at the cellular level in immune cells revealed that in CD4^+^ T cells, the m.7778G > T variant led to a higher ratio of glycolysis to OXPHOS respiration with no obvious mitochondrial functional changes, i.e., no changes in superoxide levels or ATP production. However, the small but significant alterations in the cellular respiratory profile may have resulted in the observed alterations in CD4^+^ T cell effector functions.

### 2.3. Differential Profile of Metabolites, Including Short Chain Fatty Acids, in Mice Carrying a Mutation in the mt-Atp8 Gene

**Figure 3:**
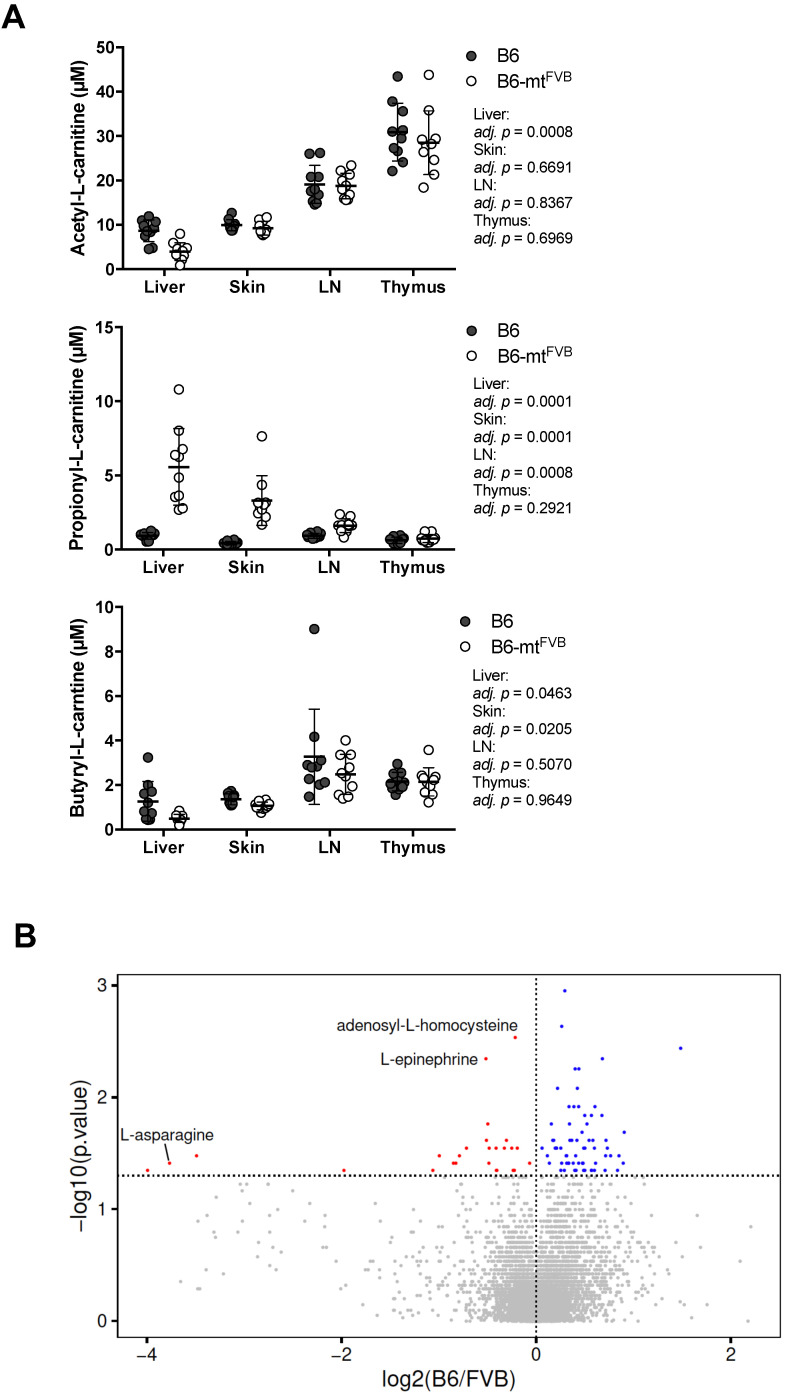

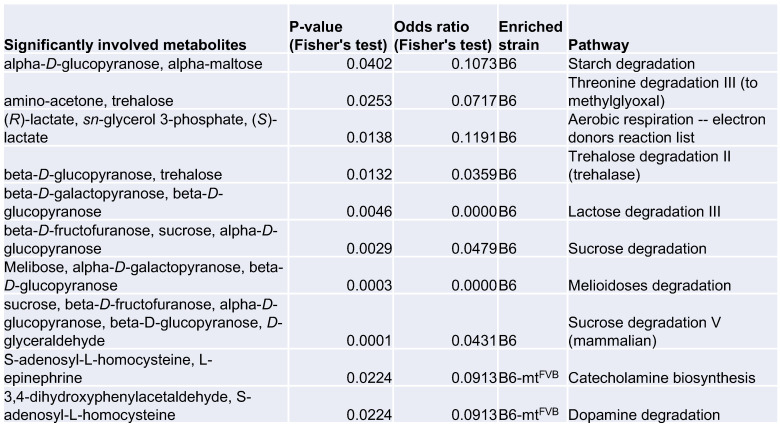
Mice carrying the m.7778G > T in the *mt-Atp8* gene demonstrated differential levels of short chain fatty acids and other metabolites compared to wild-type mice. (**A**) L-carnitine bound short chain fatty acids (acetylate, propionate, and butylate) were measured in different tissues (liver, skin, lymph nodes, and thymus) from B6-mt^FVB^ and B6 mice, by the targeted metabolomics approach. Acetyl-L-carnitine levels in the liver tissue from B6-mt^FVB^ were significantly higher than those from B6 mice (adj. *p* = 0.0008; multiple t test). Propionyl-L-carnitine levels were significantly higher in the liver, skin, and lymph node samples from B6-mt^FVB^ mice compared to those from B6 mice (adj. *p* = 0.0001, liver and skin; adj. *p* = 0.0008, lymph node; multiple t test). Butyryl-L-carnitine levels in the liver and skin samples from B6-mt^FVB^ mice were significantly higher than those from B6 mice (adj. *p* = 0.0463 and adj. *p* = 0.0205, respectively; multiple t test). LN: lymph nodes. *N* = 10 (5 males and 5 females)/strain. (**B**) An untargeted metabolomics analysis was performed in liver samples obtained from B6-mt^FVB^ and B6 mice using high-resolution ultra-high performance liquid chromatography tandem mass spectrometry (UHPLC-MS/MS). The volcano plot demonstrates the differentially identified metabolites between B6-mt^FVB^ and B6 mice. The *y*-axis indicates *p* value determined by Wilcoxon test, and the *x*-axis demonstrates the calculated log fold changes of the mean peak-area-values between the strains. *N* = 16 (6 males and 10 females)/strain, 2 to 4 months old. (**C**) Pathway enrichment analysis of the liver untargeted metabolomics dataset in Figure 3B was performed. The list shows metabolite hits in identified differentially involved metabolic pathways between B6-mt^FVB^ and B6 mice. Two pathways involving adenosyl-L-homocysteine was significantly enriched in B6-mt^FVB^ mice compared with B6 mice, while sugar degradation pathways were significantly less in B6-mt^FVB^ than B6 mice.

Mitochondria are regulatory hubs of metabolism, specifically, e.g., of the TCA cycle and lipid metabolism. We first verified our previously published data on targeted metabolomics in several peripheral tissues, namely the liver, lymph node, skin, and thymus, in B6-mt^FVB^ and B6 mice [36]. Of the identified metabolites, the levels of the short chain fatty acids (SCFAs) acetic acid, propionic acid, and butyric acid varied between B6-mt^FVB^ and B6 mice (Figure 3A). The most prominent difference between the strains was found in the propionic acid-related metabolite propionyl-L-carnitine, which was found at significantly higher levels in liver, skin, and lymph node samples from B6-mt^FVB^ mice than in the respective tissue samples from B6 mice (Figure 3A). We further evaluated the metabolite profile of liver samples from B6-mt^FVB^ and B6 mice by an untargeted metabolomics approach using LC-MS methodology. Differential abundances between the strains were identified for a number of metabolites (Figure 3B), including those predicted to be adenosyl-L-homocysteine, L-asparagine, and L-epinephrine, which were significantly enriched in B6-mt^FVB^ mice compared to B6 mice (Table S2). Pathway enrichment analysis of these metabolites revealed that two pathways involving adenosyl-L-homocysteine were significantly enriched, while sugar-degradation pathways were significantly down-regulated in B6-mt^FVB^ mice compared with B6 mice (Figure 3C). Integrated data analysis of these untargeted metabolomics data with gene array data from liver samples that we previously published [36] revealed that metabolic pathways associated with sugar degradation were enriched in liver samples from B6 mice and that pathways involved in dopamine and catecholamine metabolism were enriched in those from B6-mt^FVB^ mice (Figure S3A,B and Supplementary Materials data S1).

### 2.4. The mt-Atp8 Variant (m.7778G > T) Results in Milder Experimental Skin Inflammation than the Wild-Type Allele, a Phenomenon Mimicked by Propionate Treatment

**Figure 4:**
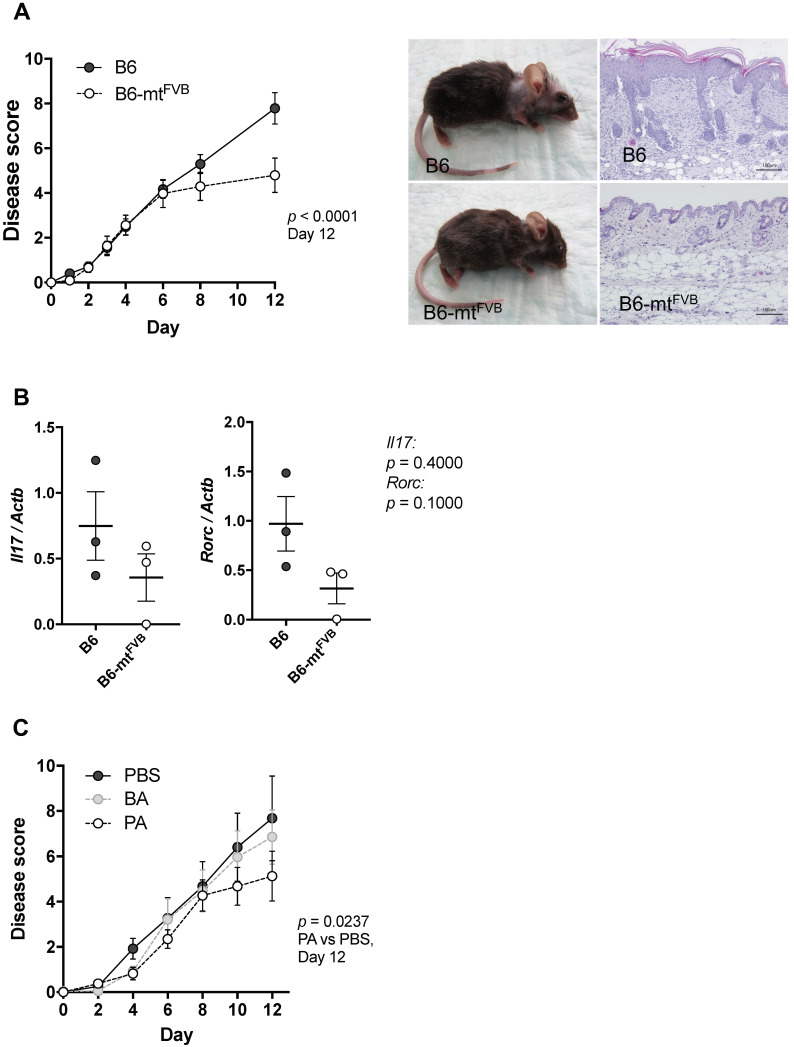
The natural variation, m.7778G > T in the *mt-Atp8* gene resulted in significantly milder disease severity in experimental skin inflammation model, and supplementation of propionic acids mimic the effect of the mutation in diseased mice. (**A**) Experimental autoimmune skin disease was induced in B6-mt^FVB^ and B6 mice by repetitive injection of pathogenic immunoglobulins G (IgG) (i.e., rabbit IgG against murine type VII collagen). Left: Disease score at day 12 in B6-mt^FVB^ mice were significantly less than that in B6 mice. *p* < 0.0001, two-way ANOVA. Right: Representative pictures of clinical phenotype and skin histopathology in B6 (top) and a B6-mt^FVB^ (bottom) mice at day 12. *N* = 20 (B6-mt^FVB^) and 17 (B6), male. (**B**) Relative expression of IL-17 and RORγT genes (*Il17* and *Rorc*, respectively) to housekeeping gene (beta-actin; *Actb*) was evaluated in neck skin samples obtained from pathogenic IgG-injected B6 and B6-mt^FVB^ mice at day 12 by qPCR. *p* = 0.4000 (*Il17*), *p* = 0.1000 (*Rorc*), Mann–Whitney *U* test, *n* = 3/strain. (**C**) Short chain fatty acids, propionic acid (PA) and butyric acid (BA), were administrated in addition to the pathogenic IgG injections in B6 mice. Mice in the control group were received PBS. Disease score in mice injected with PA exhibited significantly less than that in control mice. *p* = 0.0237, two-way ANOVA. *N* = 8 (PA) or 9 (BA and PBS). Male, 3 to 4 months old.

We next asked whether the observed alteration in immune cell functions, primarily in CD4^+^ T cells, caused by the *mt-Atp8* gene variant is linked to differential susceptibilities to experimental inflammation in mice. To answer this question, we applied a well-defined models of autoantibody-induced skin inflammation by injecting pathogenic anti-type VII collagen immunoglobulins G (IgG) into B6-mt^FVB^ and B6 mice. This induces an antibody-transfer autoimmune skin inflammation, a well-established model of epidermolysis bullosa acquisita (EBA). EBA is a variant of pemphigoid disease caused by autoantibodies directed to type VII collagen at the dermal-epidermal junction. At the end of the experiment on day 12, the disease severity, determined as the percentage of the body surface area affected by lesions, was significantly milder in B6-mt^FVB^ mice than that in B6 mice (Figure 4A,B). This phenomenon of milder disease severity in B6-mt^FVB^ mice than that in B6 mice was also observed in a mouse model of psoriasis plaque, i.e., imiquimod-induced psoriasiform dermatitis (Figure S4A,B).

There is growing evidence that microbial metabolites, including SCFAs, modulate autoimmune disease in patients and experimental models [37,38]. As shown in Figure 3A, propionate was significantly enriched in tissues from B6-mt^FVB^ mice compared to those from B6 mice. Therefore, we hypothesized that these higher propionate levels in B6-mt^FVB^ mice may have caused the reduction in disease severity of experimental autoantibody transfer autoimmune skin inflammation in these mice. B6 mice were intraperitoneally injected with either propionate, butyrate, or PBS daily for 7 days prior to disease induction by injection of pathogenic anti-type VII collagen IgG. The mice that received propionate exhibited significantly milder disease severity than those mice that received PBS (Figure 4C). Interestingly, the disease progression pattern in propionate-treated mice was similar to that in B6-mt^FVB^ mice. This finding supports the notion that the observed reduction in disease severity in B6-mt^FVB^ mice may be linked to higher levels of propionate.

## 3. Discussion

Despite the accumulating evidence for the clinical relevance of genes encoded by mitochondrial DNA, they are still understudied compared to their counterparts in the nuclear genome. There are three classes of clinically relevant mtDNA variations in humans: (1) recent maternally inherited deleterious mutations, (2) ancient adaptive polymorphisms, and (3) somatic mutations that accumulate during development and in tissues with age [39]. While adaptive mtDNA variants are commonly used to establish haplogroup ancestry, deleterious mtDNA mutations have been shown to cause rare mitochondrial disorders, such as LHON and myoclonic epilepsy with ragged red fibres (MERRF) [1,40]. In addition, several ancient polymorphisms in mtDNA have been associated with an increased risk for more common diseases, including chronic inflammation and autoimmunity [2]. Yet, the pathogenic significance of these associations and possible mechanisms in such diseases remain largely elusive.

Mitochondria, as central hubs of cellular metabolism in immune cells, have gained growing scientific attention in the field of immunology in recent years. T cell proliferation and effector function involve mitochondria-dependent signalling pathways. For example, the differentiation of T cells is accompanied by changes in mitochondrial organization and function [21], and mitochondrial reactive oxygen species are indispensable for T cell differentiation [12]. T cell activation and differentiation are tightly linked to T cell metabolism, and distinct T cell subsets depend on specific metabolic conditions [41]. Interfering with mitochondrial homeostasis by remodelling of mitochondrial membranes affects both T cell metabolism and effector function [18]. mtDNA is involved in immune function, as their role as damage-associated molecular patterns (DAMPs) in the extracellular environment causes NLR family pyrin domain containing 3 (NLRP3) inflammasome activation, resulting in proinflammatory cytokine production [42]. While many studies have focused on elucidating the inflammatory potential of mtDNA in the extracellular environment in a given inflammatory model, very few and limited studies have assessed the functional consequences of mtDNA variants in inflammation. Only one study of the functional relevance of a single mtDNA variant in immune cells has been conducted to date [14]. Therefore, we aimed to investigate the molecular impact of a single nucleotide polymorphism, m.7778G > T, in the *mt-Atp8* gene on mitochondrial functions, immune cell phenotype, culminating immune-related disease phenotypes, using our unique resource, conplastic mouse strains.

To our surprise, the polymorphism had a mild impact on the OXPHOS respiration in liver mitochondria from B6-mt^FVB^ and B6 mice, only. However, at the cellular level, we observed clear changes in the cellular metabolic profiles of CD4^+^ T cells, primarily in the levels of glycolysis; the ratio of glycolysis to OXPHOS respiration was significantly higher in cells from B6-mt^FVB^ than in those from B6 mice. These changes in cellular metabolism were underlined by differential changes in the metabolic pathways of glucose degradation and the threonine degradation, which were downregulated in B6-mt^FVB^ mice compared to B6 mice. These cellular metabolic shifts may reduce the proliferation of CD4^+^ T cells and suppress their differentiation into Th17 cells by mechanisms yet-to-be defined. The differentiation of Th17 cells, which balances Treg cell polarization, is dependent on the activation of sterol and fatty acid synthesis pathways, which produce metabolites that can act as natural agonists of the transcription factor RORγt [22,43].

In parallel, metabolomics analysis showed systemic changes in liver samples from B6-mt^FVB^ mice compared to those from B6 mice, including differential levels of short chain fatty acids, acetate, propionate and butyrate. Of these SCFAs, the levels of propionate in the liver, skin and lymph nodes were found to be elevated in B6-mt^FVB^ mice compared to B6 mice. This finding is in line with our previously published data on the gut microbiota profile of B6-mt^FVB^ mice, which identified the potent propionate-producing bacteria, Lachnospiracea [44], as an indicator species in B6-mt^FVB^ mice [45]. It is, therefore, tempting to speculate that the differential levels of SCFAs may be partly caused by a differential gut microbial composition, as previously reported [46,47]. The effects of different lipid metabolites on T cells are diverse and T cell subpopulation-specific; for instance, propionate has been shown to promote Treg differentiation, whereas saturated mid- to long-chain fatty acids (LCFAs) drive the differentiation of Th1 and Th17 cells [38].

Considering the cellular metabolic shift in CD4^+^ T cells and changes in systemic metabolite abundances identified in this study, we further investigated whether these changes cause differential clinical phenotypes in experimental diseases. We examined prototypical mouse models of inflammatory conditions in the skin, specifically, a model of the autoimmune skin blistering disease epidermolysis bullosa acquisita (EBA) and a model of plaque psoriasis. In both models, we showed that the m.7778G > T variant of the *mt-Atp8* gene resulted in milder severity of skin inflammation. Experimental EBA depends on the release of the cytokine IL-17 [48], and the induction of the IL-17 is also characteristic of the pathogenesis of psoriasis [49]. Thus, a diminished IL-17^+^ T cell population in the *mt-Atp8* mutant mice likely contributed to the decrease in disease severity in both skin inflammation models in this study. Interestingly, it was previously reported that propionate reduces Th17 differentiation [38], suggesting a potential link between our identified metabolic changes (i.e., increase in propionyl-carnitine levels) and diminished Th17 differentiation in B6-mt^FVB^ mice. SCFAs, particularly acetate, propionate, and butyrate, have been highlighted in recent years to exert immunomodulatory effects through multiple mechanisms, in a manner mediated by G-protein coupled receptors (GPCRs), nuclear receptors, and epigenetic changes, among others [50]. Propionate, for instance, perturbs liver (mitochondrial) metabolism [51], induces apoptosis in neutrophils [52], and influences haematopoiesis and airway inflammation [53]. The net effects of the actions of these SCFAs are context-dependent and can be pro- or anti-inflammatory. Most intriguingly, it has recently been shown that oral administration of propionate to experimental autoimmune encephalomyelitis model mice and to multiple sclerosis patients significantly abrogates disease [37,38]. Similarly, we demonstrated here that administration of exogenous propionate reduced autoimmune skin inflammation in wild-type mice. These reports and our findings encourage further investigation of the therapeutic effect of propionate in patients suffering from autoantibody-driven diseases.

An open question remains: How are mitochondrial gene polymorphisms associated with metabolic changes? The m.7778G > T variant in the *mt-Atp8* gene leads to a change from aspartate to tyrosine at position 5 of the ATP8 protein (A6L). Previously, it was reported that deficiency of MT-ATP6/8 in human cybrid cells destabilizes complex V assembly and oligomerization, causing dysfunction in complex V, decreased respiration, and increased glycolysis [54]. This implies an altered inner mitochondrial membrane organization and is predicted to affect OXPHOS efficiency [55,56,57], indicating the functional relevance of the ATP8 gene to the ATP synthase. The polymorphism m.7778G > T in the *mt-Atp8* gene leads to relatively higher activity levels in complex V, but the change is not sufficient to cause dramatic changes in OXPHOS function, as the gene variant does not result in a loss of function and is likely to be counteracted by mitochondrial homeostasis mechanisms. To maintain a balance in energy production, subtle changes in the OXPHOS machinery may be controlled by restricting the supply of the electron carrier NADH and/or FADH_2_ into the OXPHOS machinery, limiting TCA cycle activity and/or substrate (e.g., pyruvate) availability. Interestingly, six of the eight pathways identified to be decreased in B6-mt^FVB^ mice compared to B6 mice by integrated analysis of metabolomics and gene expression data are related to the degradation of various sugars, suggesting broad changes in the regulation of glycolysis in B6-mt^FVB^ mice. This is in line with our finding of relatively increased glycolysis in CD4^+^ T cell, as a putative compensatory mechanism for maintaining energy homeostasis. Furthermore, the threonine degradation pathway, another pathway that was found to be decreased in B6-mt^FVB^ mice compared to B6 mice, involves the production of NADH from NAD^+^ via threonine dehydrogenase. Threonine degradation may proceed towards propionyl-CoA/succinate or methylglyoxal, which is also a glycolysis end product and has a wide range of effects on cellular function, as well as towards other metabolites [58,59]. These complex changes and their interactions could be potential causes of the hindered effector functions of CD4^+^ T cells in B6-mt^FVB^ mice compared to those in B6 mice, i.e., less proliferation upon the T cell stimulation and IL-17 production. Furthermore, such changes in metabolites could alter the microenvironment in the gut, which is tightly linked with the gut microbiota, and consequently alter the composition of microbial metabolites, such as SCFAs. It has been reported that the specific tissue micromilieu affects mitochondrial metabolism and can suppress T cell function [60]. It is therefore conceivable that significant changes in the skin metabolite profile are likely to further impact mitochondrial functions in immune cells from B6-mt^FVB^ mice. Modification of cell metabolism is becoming a powerful tool for the modulation of autoimmune diseases. Propionate and other metabolic interventions have been shown to reverse the disease phenotype of lupus erythematosus and biomarker levels by suppressing cytokine production in T cells [61].

In summary, this study provides evidence that a maternally inherited adaptive mtDNA polymorphism that leads to a very mild change in OXPHOS function causes alterations in the systemic metabolite profile. Consequently, changes in metabolites, such as elevated propionate levels, are associated with the modulation of CD4^+^ T cell effector functions through changes in cellular metabolism. Both cellular and systemic alterations in metabolites are linked to the subsequent and significant changes in the course of autoimmune and autoinflammatory tissue inflammation in mice. In addition, we provide experimental evidence of the potential of metabolic intervention, i.e., the administration of metabolites that are found at different levels as a result of mtDNA variants, to treat inflammatory diseases. Based on the results presented in this study, further exploration of the impact of adaptive mtDNA variants on tissue-specific metabolic profiles (e.g., that of the inflammatory skin microenvironment), as well as immunometabolic modulation in other immune cell subpopulations involved in complex inflammatory diseases, is warranted.

## 4. Materials and Methods

### 4.1. Mouse Husbandry

The conplastic mouse strain, C57BL/6J-mt^FVB/NJ^ (B6-mt^FVB^), was previously generated (Yu et al., 2009). C57BL/6J (B6) mice (Stock No: 000664) were purchased from Jackson Laboratory (Bar Harbor, ME, USA) and bred in the animal facility of University of Luebeck. The mutations in the mtDNA of the B6-mt^FVB^ and B6 mice are listed in Table S1. Genotyping of nuclear genome of both B6-mt^FVB^ and B6 were conducted using the MegaMUGA Mouse Universal Genotyping Array (77,808 SNPs) as described previously [6]. Greater than 99.9% of SNPs in B6-mt^FVB^ mice were identical to those of B6 mice (Table S3). The B6-mt^FVB^ strain was maintained by repeated backcrossing female B6-mt^FVB^ with male B6 mice, which were randomly selected from the B6 colony maintained in the same breeding facility room.

The animal facility was maintained at 21 °C on a 12 h light-12 h dark-cycle. Mice had ad libitum access to filtered water and pellet diet (1314, Altromin, Lage, Germany). Animal use and procedures were approved by local authorities of the Animal Care and Use Committee (Kiel, Germany) and performed by certified personnel.

### 4.2. Isolation of Liver Mitochondria

Liver tissue is homogenized with Potter-Elvehjem glass homogenizer (size 22: 100–150 µm clearance; (Kimble Chase, Rockwood, TN, USA) in with 5 mL ice-cold mitochondria isolation buffer (0.2 mM EDTA, 0.25 M sucrose, 10 mM Tris-HCl pH 7.8) supplemented with 1× Halt^TM^ Protease & Phosphatase Inhibitor (Thermo Fisher Scientific, Darmstadt, Germany). Homogenates were centrifuged at 1000 *g* for 10 min at 4 °C to pellet the cells debris and nuclei. The supernatant was centrifuged again at 1000 *g* for 10 min at 4 °C to remove remaining debris. The supernatant was then centrifuged at 10,000 *g* for 15 min at 4 °C to pellet the mitochondrial fraction. The pellet was washed in fresh mitochondria-isolation buffer and pelleted again by centrifugation at 10,000 *g* for 15 min at 4 °C. Finally, mitochondrial enriched pellet was suspended in mitochondria isolation buffer, and protein concentration was determined by BCA assay according to standard procedures (Pierce BCA Protein Assay Kit, Thermo Fisher Scientific, Mannheim, Germany).

### 4.3. Determination of ATP and Mitochondrial ROS Levels in Isolated Mitochondria

To evaluate ATP production kinetics, isolated liver mitochondria were diluted in 100 µL ice cold assay buffer (125 mM KCl, 2 mM K_2_HPO_4_, 20 mM HEPES, 1 mM MgCl_2_, 100 µM EGTA, 0.025% BSA, pH 7.0) supplemented with 2.5 mM malate, 2.5 mM glutamate, 2.5 mM succinate, and 3 mM ADP. Control reactions were additionally supplemented with 2.5 µM oligomycin to inhibit ATP synthase activity and 2.5 µM FCCP to uncouple respiration and ATP synthase activity. Samples were incubated for 30 min at 37 °C. Afterwards, to detect the generated ATP content, the reaction mix was supplemented with 100 µL CellTiter-Glo Luminescent Cell Viability Assay reaction buffer (Promega, Mannheim, Germany). Chemiluminescence was detected using the Infinite M200 PRO ELISA reader (Tecan Deutschland GmbH, Crailsheim, Germany).

Mitochondrial superoxide was determined using MitoSOX Red (Invitrogen, Thermo Fisher Scientific, Schwerte, Germany). Reactions were supplemented with 200 nM MitoSOX Red. Reactions were incubated at 37 °C and MitoSOX Red fluorescence was evaluated at the following wavelength settings: excitation: 510 nm and emission: 580 nm on the Infinite M200 PRO ELISA reader (Tecan Deutschland GmbH, Crailsheim, Germany).

Hydrogen peroxide levels in liver mitochondria were measured using Amplex Red HRP kit (Invitrogen, Thermo Fisher Scientific, Schwerte, Germany), according to the manufacturer’s protocol.

### 4.4. OXPHOS Enzyme Activity Measurement

In vitro measurements of OXPHOS enzymes in liver mitochondria were performed as previously described [7].

### 4.5. Western Blot Analysis

Western blot analysis was performed according to standard protocols. Liver mitochondria were lysed with RIPA buffer, and the supernatant was used for Western blotting as previously described [7].

### 4.6. mtDNA Copy Number

Liver DNA was used to amplify and quantify mitochondrial encoded genes (*mt-Nd5* and *mt-Co1*) and nuclear encoded genes (*Actb*) by qPCR. The analysis was performed according to the previously published protocol [62].

### 4.7. Immune Cell Preparation

Mouse lymph nodes and spleens were disrupted by mechanical shearing, cells were passed through a 70 µm filter and residual erythrocytes were disrupted by 5 min incubation in red blood cell (RBC) lysis buffer. After washing, lymphocytes and splenocytes were cultured in RPMI1640 supplemented with 2 mM L-Glutamine, 1 mM Na-Pyruvate, 1× minimum essential medium (MEM) non-essential amino acids, 100 U/mL Penicillin and 100 µg/mL Streptomycin, 10% foetal bovine serum (FBS), and 50 µM 2-Mercaptoethanol (2-ME). T cells were activated with plate-bound anti-mouse CD3 antibody (1 µg/mL; clone 145-2C11) and soluble anti-mouse CD28 antibody (0.2 µg/mL; clone 37.51) for 48 h. In experiments for intracellular cytokine staining, cells were incubated for 4 h with monensin before proceeding with cell fixation/permeabilization and intracellular staining of cytokines.

CD4^+^ T or B cells were enriched by negative selection in the magnetic field using CD4^+^ T Cell Isolation Kit or B Cell Isolation Kit (Miltenyi Biotech B.V. & Co. KG, Bergisch Gladbach, Germany) and LS Column, according to the manufacturer’s protocol.

### 4.8. Flow Cytometry Analysis

Intracellular staining of cytokines: After 48 h T cell stimulation, cells were incubated with phorbol 12-myristate 13-acetate (PMA) and monensin for 4 h, cells were washed in PBS and suspended in 2% FBS/PBS and 10 µg/mL anti-mouse CD16/CD32 antibody to block unspecific antibody binding. Cells were incubated for 10 min on ice with anti-CD4-APC or anti-CD4-PerCP-eFluor710 (both clone: RM4-5), and anti-CD25-PerCP-Cy5.5 (clone: PC61.5) antibodies. After washing, the cells were fixed and permeabilized using the eBioscience FoxP3/Transcription Factor Staining Buffer Set (Thermo Fisher Scientific, Darmstadt, Germany). After initial fixation for 1 h at room temperature cells were washed twice with Permeabilization buffer. Fixed cells, suspended in Permeabilization buffer were incubated with anti-IFN-gamma- FITC (clone: XMG1.2), anti-IL-4-APC (clone: 11B11), and anti-IL-17A-PE (eBio17B7) for 20 min on ice. Cells were washed once more with Permeabilization buffer and suspended in 2% FBS/PBS for flow cytometry analysis using a FACSCalibur (BD, Heidelberg, Germany). All antibodies were purchased from (eBioscience/Thermo Fisher Scientific, Darmstadt, Germany).

BrdU proliferation assay: After stimulation of lymphocytes with anti-mouse CD3 and anti-mouse CD28 antibodies for 48 h, 10 µM BrdU (Sigma, Taufkirchen, Germany) was added to the cultured cells and incubated overnight. Cells were washed in PBS and suspended in 2% FBS/PBS and 10 µg/mL anti-mouse CD16/CD32 antibody to block unspecific antibody binding. Cells were incubated for 15 min on ice with anti-mouse CD4-APC (clone: RM4-5) and anti-mouse CD8-eFluor710 (clone: 53–6.7) and anti-mouse CD25-PerCP-Cy5.5 (clone: PC61.5) antibodies. After washing, the cells were fixed and permeabilized using the BD Cytofix/Cytoperm Kit (BD Biosciences, Heidelberg, Germany). Fixed/permeabilized cells were treated with 300 µg/mL DNase I for 1 h at 37 °C. After washing, cells were incubated with anti-BrdU-FITC (clone: 3D4) antibodies for 20 min, washed again and suspended in 2% FBS/PBS for flow cytometry analysis.

### 4.9. Mitochondrial Superoxide and Mitochondrial Membrane Potential in CD4^+^T Cells

Primary lymphocytes were measured immediately (basal levels) and after 24 h culture with anti-mouse CD3 and anti-mouse CD28 antibodies (T cell activation). Cells were washed in 2% FBS/PBS and labeled with 5 µM MitoSOX Red^TM^ for 10 min at 37 °C or 150 nM tetramethylrhodamine, ethyl esther, perchlorate (TMRE) and 100 nM MitoTracker Green FM for 15 min at 37 °C. Cells were washed again with 2% FBS/PBS and analysed by flow cytometry on a FACSCalibur.

### 4.10. ATP Content in CD4^+^ T Cells

To determine the ATP content in CD4^+^ T cells, 1 × 10^5^ CD4^+^ T cells were suspended in 100 µL medium (4 mM L-glutamine, 25 mM glucose, 1 mM Na-pyruvate in Dulbecco’s Modified Eagle’s Medium (DMEM) without phenol red, pH 7.4) and supplemented with 100 µL of the reaction reagent of the CellTiter-Glo^®^ Luminescent Cell Viability Assay Kit (Promega, Mannheim, Germany). ATP dependent chemiluminescence was determined on the Infinite M200 PRO ELISA reader (Tecan Deutschland GmbH, Crailsheim, Germany) and compared between wildtype and B6-mt^FVB^ samples.

### 4.11. Cellular Metabolism Flux Assay Using Seahorse Bioanalyzer

Using the Seahorse XF24 Extracellular Flux Analyzer (Agilent Technologies, Waldbronn, Germany), it was possible to measure cellular flux processes by measuring the oxygen and proton concentration in the medium. Addition of inhibitors and chemical modulators (i.e., inhibitors and uncoupler) enables the differentiation of the respiratory chain activity and determines mitochondrial oxygen respiration function, as well as glycolytic flux, as determined by extracellular acidification through lactate coupled proton exocytosis [63,64,65]. In preparation, XF 24-well microplates were coated with CellTak (Corning, Corning, NY, USA) to facilitate attachment of suspension cells to the plate surface. CellTak was diluted to 22 µg/mL in 0.1 M Na-bicarbonate buffer (pH 8.0) and incubated for a minimum of 20 min to ensure sufficient binding of the protein mix followed by 2 washing steps with distilled water. Cells were seeded at 1.5 × 10^6^ cells per well in 525 µL XF assay medium (4 mM L-glutamine, 25 mM glucose, 1 mM Na-pyruvate in DMEM without phenol red, pH 7.4). Plates were centrifuged shortly at 40 *g* and again at 80 *g* to quickly attached cells to the CellTak-coated culture surface. Cells were incubated for 1 h at 37 °C without additional CO_2_. Injection ports were filled each with 75 µL XF assay medium containing pre-diluted oligomycin (final concentration in assay 1 µM) or FCCP (final concentration in assay 1.2 µM) or antimycin A and rotenone (final concentration in assay each 1 µM), or 2-Deoxyglucose (2-DG; 100 mM in the assay), respectively, to differentiate various respiration factors in the assay.

### 4.12. Neutrophil Granulocyte Functional Phenotyping

Bone marrow was flushed out from femur and tibia of mice. Neutrophil granulocytes were isolated by Percoll differential centrifugation [66]. Cells were kept in Hank’s Balanced Salt Solution (HBSS) containing 20 mM HEPES and 0.5% FCS and were stimulated with 200 nM PMA (phorbol 12-myristate 13-acetate) for 30 min. Oxidative burst was measured with DHR123 after incubation for 30 min. Surface marker staining was performed using anti-mouse CD62L-FITC, anti-mouse CD11b-FITC, and anti-mouse CD69-PE antibodies. Spontaneous apoptosis was measured in unstimulated neutrophils after 42 h of incubation in RPMI medium supplemented with 10% FCS and non-essential amino acids followed by staining with Annexin V and propidium iodide (PI).

### 4.13. Untargeted Metabolomics

Tissues sample were collected and frozen in liquid nitrogen and stored at −80 °C until extraction. Isolated cells were washed in PBS and frozen in liquid nitrogen and stored at −80 °C until extraction. One hundred to two hundred milligrams of tissues were used for isolation. Procedures were performed on ice. Pre-weighed samples were transferred into plastic tubes containing glass beads and 4 µL/mg of methanol, and 0.85 µg/mg of water were added. Samples were homogenized using a Speedmill PLUS (Analytik Jena, Jena, Germany). The homogenized mixture was transferred into clean glass vials 1 µL/mg of methanol/water (1:0.8) was added to the plastic tube, vortexed for 15 s, and the remaining material was transferred the samples in the glass vials. Next, 5 µL/mg of chloroform and 2 µL/mg water were added the glass vials, followed by 30 s of vortexing and centrifugation at 1800× *g* for 10 min at 4 °C. The samples were left at room temperature for 5 min. The upper (polar) and lower (non-polar) phases were separated, and a defined volume was added into fresh glass vials. The contents were dried under nitrogen stream at room temperature and stored until analysis. Dried samples were reconstituted in 900 µL of 20% of mobile phase B and 100 µL chloroform and centrifuged. Three microliters were injected into the ultra-high performance liquid chromatography-tandem mass spectrometry (UHPLC-MS/MS) system.

Lipids were then directly analyzed using a Vanquish UHPLC-System (Thermo Scientific, Waltham, MA, USA) with a heated electrospray ionization (HESI) QExactive plus Orbitrap mass spectrometer (Thermo Scientific, Waltham, USA). Chromatographic separation took place on a reversed-phase column (Accucore Polar Premium 100 × 2.1 mm (2.6 µm) with guard column: Accucore Polar Premium 10 × 2.1 mm (2.6 µm); Thermo Scientific, Waltham, MA, USA). Autosampler temperature was 10 °C throughout the whole measurements.

Mobile phase A consisted of 60% acetonitrile, 10 mM ammonium formate and 0.1% formic acid in ultrapure water. Mobile phase B was 90% isopropanol, 10 mM ammonium formate, and 0.1% formic acid in ultrapure water. Separation was performed with an increasing gradient of B (20–100% from 0.5 to 8.5 min and 20% from 12.5 to 15 min) over a total time of 15 min. Flow rate employed was 0.4 mL/min, and the column temperature was 55 °C. The analytes were detected by a Thermo Orbitrap mass spectrometer equipped with a HESI source operated in the positive ion mode. MS data were acquired over a scan range of 250–1200 m/z with full MS resolution of 70,000 and data-dependent MS^2^ resolution of 17,500.

Methanol extracts: Chromatographic separation was done on a reversed-phase column (Accucore Polar Premium 100 × 2.1 mm (2.6 µm) with guard column: Accucore Polar Premium 10 × 2.1 mm (2.6 µm); Thermo Scientific, Waltham, MA, USA). Mobile phase A consisted of ultrapure water with 0.1% formic acid. Mobile phase B was methanol with 0.1% formic acid. Separation was performed with an increasing gradient of B (1–10% from 0.5 to 2 min, 10–99% from 2 to 10.5 min, and 1% from 12.1 to 15 min) over a total time of 15 min. Flow rate employed was 0.3 mL/min from 0–10.5 min and 0.4 mL/min from 12–14.9 min. MS data were acquired over a scan range of 100–1500 m/z with full MS resolution of 70,000 and data-dependent MS^2^ resolution of 17,500.

Further chromatographic separation was performed on an Amid HILIC (100 × 2.1 mm) with guard column: Amid HILIC 10 × 2.1 mm; Thermo Scientific, Waltham, MA, USA). Mobile phase A consisted of 50% acetonitrile with 10 mM AF and 0.1% formic acid in ultrapure water and mobile phase B was 95% acetonitrile with 10 mM AF and 0.1% formic acid in ultrapure water (positive ion modus). Mobile phase A consisted of 50% acetonitrile with 10 mM AA and 0.1% formic acid in ultrapure water, and mobile phase B was 95% acetonitrile with 10 mM AA and 0.1% formic acid in ultrapure water (negative ion modus). Separation was performed with a decreasing gradient of B 99–85% from 1 to 3 min, 85–50% from 3 to 6 min, and 50–5% from 6 to 9 min) over a total time of 14 min. Flow rate employed was 0.5 mL/min. MS data were acquired over a scan range of 70–1050 m/z with full MS resolution of 70,000 and data-dependent MS^2^ resolution of 17,500.

Identification and quantification of individual lipid species were performed by LipidSearch Software from Thermo Scientific (Waltham, MA, USA) on product level (MS/MS fragmentation).

The analytes were detected by a Thermo Orbitrap mass spectrometer equipped with a HESI source operated in the positive and negative ion mode.

### 4.14. An Integrated Analysis of Untargeted Metabolomics Data and Gene Array Expression Data

Following untargeted metabolomic measurements, metabolite identities were predicted by matching the measured mass-charge-ratios (m/z) to the expected m/z values of adducts from metabolites, which are described as part of the mouse metabolome using a mass accuracy window of 10 ppm. As reference *Mus musculus* metabolites, the MouseCyc resource [67] (Version 24.0), which is now integrated in the BioCyc database collection [68], was used. Wilcoxon rank sum tests with continuity correction were performed to determine significant differences in individual metabolite levels between samples from B6 and B6-mt^FVB^ mice.

Identified metabolites with significant differences between the two mouse strains were subjected to subsequent pathway enrichment analysis based on metabolic pathways as defined in the MouseCyc database. Pathways were considered as enriched if more metabolites displayed significantly higher levels in one genotype over the other than expected by chance. Significance of the enrichment was statistically assessed using the Fisher’s exact test for count data. The MouseCyc metabolic network database comprises, in addition to the information on metabolites and metabolic reactions, the information on which *Mus musculus* genes are associated to certain reactions within the metabolic network. This gene-reaction-metabolite network was employed in order to determine which differentially expressed genes are associated with reactions that involve metabolites for which differences in their levels were observed in the metabolomics data. Interactions of differentially expressed genes and metabolites with significant differences between the mouse strains were visualized using the R-package *igraph* [69] for Figure S3.

The above-described pathway and network analysis of the untargeted metabolomics and gene array data was performed in R (version 3.6.3). The R-code to reproduce results shown in Figure 3B, Figure S3, and Table S2 is available at the public github repository (https://github.com/Waschina/atp8_metabopwy).

### 4.15. Autoantibody-Transfer Experimental Autoimmune Skin Inflammation (Epidermolysis Bullosa Acquisita; EBA) Model

Antibody-induced experimental autoimmune skin inflammation was induced according to the previously described protocol [34] with a modification. More specifically, mice were subcutaneously injected with 3 mg of pathogenic IgG, i.e., total rabbit IgG against murine type VII collagen IgG (“anti-type VII collagen IgG”) on day 0 and day 2 of the experiment. Disease severity was scored as the percentage of skin surface area affected by erythema, blisters, erosion, crusts, or alopecia [34]. Back skin samples were stored in 4% Histofix solution (Carl Roth GmbH, Karlsruhe, Germany) for histopathology or in RNAlater (Thermo Fisher Scientific, Schwerte, Germany) for the RNA extraction.

In separate experiments mice were injected intraperitoneally with either 1 g/kg/day propionate (PA), butyrate (BA), or PBS in the control group. The daily injections of PA, BA, or PBS were starting 7 days before disease induction through subcutaneous injection of the affinity-purified anti-type VII collagen IgG (50 µg/mouse) on day 0, day 2, and day 4. Disease severity was scored as described above.

### 4.16. Imiquimod (Aldara^®^)-Induced Psoriasisform Dermatitis (AIPD) Model

Psoriasiform dermatitis was induced by applying 50 mg of Aldara cream (MEDA Pharma), including 5% imiquimod (IMQ) on the shaved and depilated dorsal skin area and ears of mice for 4 consecutive days. The severity of resulting dermatitis was scored daily using a modification of the Psoriasis Activity and Severity Index (PASI), evaluating the extend of erythema, infiltration, and desquamation on a scale from 0 to 4, as previously described [35,70]. Back skin samples were stored in 4% Histofix solution or RNAlater, as performed in autoantibody-induced skin inflammation model.

### 4.17. Histopathology

Skin samples fixed in 4% Histofix solution (Carl Roth) were processed into paraffin blocks. Six micrometer sections were prepared and stained with haematoxylin and eosin according to standard protocol.

Staining was visualized and photographed using the BZ-9000E series Keyence microscope (Keyence GmbH, Neu-Insenburg, Germany). BZ-II Analyzer software (Keyence) was used to measure epidermal thickness, i.e., the length between the dermal-epidermal junction and the surface of the epidermis in IMQ-induced psoriasiform dermatitis mice. The average value (µm) of 8 different spots in a ×100 magnified image of the section per mouse was taken for the analysis.

### 4.18. Quantitative Real-Time PCR

Total RNA was isolated from the RNAlater-stored tissues using the QIAzol (Qiagen), according to the manufacturer’s protocol. After reverse transcription using First Strand cDNA synthesis Kit (Thermo Fisher Scientific, Schwerte, Germany) the cDNA was added to the Maxima SYBR Green qPCR Master Mix (Thermo Fisher Scientific, Schwerte, Germany) and amplified using RealPlex thermal cycler (Eppendorf, Hamburg, Germany). The amplification program consists of initial denaturation 95 °C for 10 min, 40 cycles of denaturation at 95 °C for 15 s, and annealing at 60 °C for 30 s, followed by extension at 72 °C for 30 s. The beta-actin gene (*Actb*) was used as a house-keeping gene. Sequences for the primers used in this study are;

*Il17* forward, 5′-tcagcgtgtccaaacactgag-3′; *Il17* reverse, 5′-cgccaagggagttaaagactt-3′

*Rorc* forward, 5′-gatctaagggctgaggcacc-3′; *Rorc* reverse, 5′-cacattacactgctggctgc -3′.

*Actb* forward, 5′-cactgtcgagtcgcgtcc-3‘; *Actb* reverse, 5′-cgcagcgatatcgtcatcca-3′.

### 4.19. Statistics

Data presented as mean and standard error of mean. Statistical analysis was performed using GraphPad Prism v6.07 (GraphPad Software,), and statistical tests used for analysis are indicated in the figure legends and/or in the text.

Statistical tests were performed only for descriptive purposes, and descriptive *p* values are reported.

## Data Availability

Data is contained within the article or supplementary material.

## Supplementary Materials

The following are available online at https://www.mdpi.com/1422-0067/22/3/1006/s1, Figure S1: Data related to Figure 1, Figure S2: Data related to Figure 2, Figure S3: Data related to Figure 3, Figure S4: Differential clinical presentation of B6-mt^FVB^ and B6 mice in Imiquimod (IMQ)-induced skin inflammation model. Table S1: List of mitochondrial genome variations in B6-mt^FVB^ and B6 mice, Table S2: List of potential metabolites differentially expressed in liver samples from B6-mt^FVB^ and B6 mice based on untargeted metabolomics, Table S3: Nuclear genome differences between B6-mt^FVB^ and B6 mice, Data S1: Networks identified in an integrative network analysis of metabolomics and gene array data sets.

## References

[1] Wallace D.C., Zheng X.X., Lott M.T., Shoffner J.M., Hodge J.A., Kelley R.I., Epstein C.M., Hopkins L.C. (1988). Familial Mitochondrial Encephalomyopathy (MERRF): Genetic, Pathophysiological, and Biochemical Characterization of a Mitochondrial DNA Disease. Cell.

[2] Wallace D.C. (2005). A Mitochondrial Paradigm of Metabolic and Degenerative Diseases, Aging, and Cancer: A Dawn for Evolutionary Medicine. Annu. Rev. Genet..

[3] Brown M.D., Starikovskaya E., Derbeneva O., Hosseini S., Allen J.C., Mikhailovskaya I.E., Sukernik R.I., Wallace D.C. (2002). The Role of MtDNA Background in Disease Expression: A New Primary LHON Mutation Associated with Western Eurasian Haplogroup. J. Hum. Genet..

[4] Lin C.S., Sharpley M.S., Fan W., Waymire K.G., Sadun A.A., Carelli V., Ross-Cisneros F.N., Baciu P., Sung E., McManus M.J. (2012). Mouse MtDNA Mutant Model of Leber Hereditary Optic Neuropathy. Proc. Natl. Acad. Sci. USA.

[5] Hashizume O., Shimizu A., Yokota M., Sugiyama A., Nakada K., Miyoshi H., Itami M., Ohira M., Nagase H., Takenaga K. (2012). Specific Mitochondrial DNA Mutation in Mice Regulates Diabetes and Lymphoma Development. Proc. Natl. Acad. Sci. USA.

[6] Hirose M., Schilf P., Gupta Y., Wright M.N., Wright M.N., Jöhren O., Wagner A.E., Sina C., Ziegler A., Ristow M. (2016). Lifespan Effects of Mitochondrial Mutations. Nature.

[7] Hirose M., Schilf P., Gupta Y., Zarse K., Künstner A., Fähnrich A., Busch H., Yin J., Wright M.N., Ziegler A. (2018). Low-Level Mitochondrial Heteroplasmy Modulates DNA Replication, Glucose Metabolism and Lifespan in Mice. Sci. Rep..

[8] Hirose M., Künstner A., Schilf P., Tietjen A.K., Jöhren O., Huebbe P., Rimbach G., Rupp J., Schwaninger M., Busch H. (2019). A Natural MtDNA Polymorphism in Complex III Is a Modifier of Healthspan in Mice. Int. J. Mol. Sci..

[9] Hirose M., Schilf P., Zarse K., Busch H., Fuellen G., Jöhren O., Köhling R., König I.R., Richer B., Rupp J. (2019). Maternally Inherited Differences within Mitochondrial Complex I Control Murine Healthspan. Genes.

[10] Latorre-Pellicer A., Moreno-Loshuertos R., Lechuga-Vieco A.V., Sánchez-Cabo F., Torroja C., Acín-Pérez R., Calvo E., Aix E., González-Guerra A., Logan A. (2016). Mitochondrial and Nuclear DNA Matching Shapes Metabolism and Healthy Ageing. Nature.

[11] Galati D., Srinivasan S., Raza H., Prabu S.K., Hardy M., Chandran K., Lopez M., Kalyanaraman B., Avadhani N.G. (2009). Role of Nuclear-Encoded Subunit Vb in the Assembly and Stability of Cytochrome c Oxidase Complex: Implications in Mitochondrial Dysfunction and ROS Production. Biochem. J..

[12] Sena L.A., Li S., Jairaman A., Prakriya M., Ezponda T., Hildeman D.A., Wang C., Schumacker P.T., Licht J.D., Perlman H. (2013). Mitochondria Are Required for Antigen-Specific T Cell Activation through Reactive Oxygen Species Signaling. Immunity.

[13] Ansó E., Weinberg S.E., Diebold L.P., Thompson B.J., Malinge S., Schumacker P.T., Liu X., Zhang Y., Shao Z., Steadman M. (2017). The Mitochondrial Respiratory Chain Is Essential for Haematopoietic Stem Cell Function. Nat. Cell Biol..

[14] Loveland B., Wang C.R., Yonekawa H., Hermel E., Lindahl K.F. (1990). Maternally Transmitted Histocompatibility Antigen of Mice: A Hydrophobic Peptide of a Mitochondrially Encoded Protein. Cell.

[15] Ishikawa K., Toyama-Sorimachi N., Nakada K., Morimoto M., Imanishi H., Yoshizaki M., Sasawatari S., Niikura M., Takenaga K., Yonekawa H. (2010). The Innate Immune System in Host Mice Targets Cells with Allogenic Mitochondrial DNA. J. Exp. Med..

[16] O’Neill L.A.J., Kishton R.J., Rathmell J. (2016). A Guide to Immunometabolism for Immunologists. Nat. Rev. Immunol..

[17] Pearce E.L., Pearce E.J. (2013). Metabolic Pathways in Immune Cell Activation and Quiescence. Immunity.

[18] Buck M.D., O’Sullivan D., Klein Geltink R.I., Curtis J.D., Chang C.-H., Sanin D.E., Qiu J., Kretz O., Braas D., van der Windt G.J.W. (2016). Mitochondrial Dynamics Controls T Cell Fate through Metabolic Programming. Cell.

[19] Klein Geltink R.I., O’Sullivan D., Corrado M., Bremser A., Buck M.D., Buescher J.M., Firat E., Zhu X., Niedermann G., Caputa G. (2017). Mitochondrial Priming by CD28. Cell.

[20] O’Sullivan D., van der Windt G.J.W., Huang S.C.-C., Curtis J.D., Chang C.-H., Buck M.D., Qiu J., Smith A.M., Lam W.Y., DiPlato L.M. (2014). Memory CD8(+) T Cells Use Cell-Intrinsic Lipolysis to Support the Metabolic Programming Necessary for Development. Immunity.

[21] van der Windt G.J.W., Everts B., Chang C.-H., Curtis J.D., Freitas T.C., Amiel E., Pearce E.J., Pearce E.L. (2012). Mitochondrial Respiratory Capacity Is a Critical Regulator of CD8+ T Cell Memory Development. Immunity.

[22] Berod L., Friedrich C., Nandan A., Freitag J., Hagemann S., Harmrolfs K., Sandouk A., Hesse C., Castro C.N., Bähre H. (2014). De Novo Fatty Acid Synthesis Controls the Fate between Regulatory T and T Helper 17 Cells. Nat. Med..

[23] Gerriets V.A., Kishton R.J., Johnson M.O., Cohen S., Siska P.J., Nichols A.G., Warmoes M.O., de Cubas A.A., MacIver N.J., Locasale J.W. (2016). Foxp3 and Toll-like Receptor Signaling Balance Treg Cell Anabolic Metabolism for Suppression. Nat. Immunol..

[24] Michalek R.D., Gerriets V.A., Jacobs S.R., Macintyre A.N., MacIver N.J., Mason E.F., Sullivan S.A., Nichols A.G., Rathmell J.C. (2011). Cutting Edge: Distinct Glycolytic and Lipid Oxidative Metabolic Programs Are Essential for Effector and Regulatory CD4+ T Cell Subsets. J. Immunol..

[25] Priyadharshini B., Loschi M., Newton R.H., Zhang J.-W., Finn K.K., Gerriets V.A., Huynh A., Rathmell J.C., Blazar B.R., Turka L.A. (2018). Cutting Edge: TGF-β and Phosphatidylinositol 3-Kinase Signals Modulate Distinct Metabolism of Regulatory T Cell Subsets. J. Immunol..

[26] Mills E.L., Ryan D.G., Prag H.A., Dikovskaya D., Menon D., Zaslona Z., Jedrychowski M.P., Costa A.S.H., Higgins M., Hams E. (2018). Itaconate Is an Anti-Inflammatory Metabolite That Activates Nrf2 via Alkylation of KEAP1. Nature.

[27] Van den Bossche J., Baardman J., Otto N.A., van der Velden S., Neele A.E., van den Berg S.M., Luque-Martin R., Chen H.-J., Boshuizen M.C.S., Ahmed M. (2016). Mitochondrial Dysfunction Prevents Repolarization of Inflammatory Macrophages. Cell Rep..

[28] Mehta M.M., Weinberg S.E., Chandel N.S. (2017). Mitochondrial Control of Immunity: Beyond ATP. Nat. Rev. Immunol..

[29] Sack M.N. (2018). Mitochondrial Fidelity and Metabolic Agility Control Immune Cell Fate and Function. J. Clin. Investig..

[30] Bayona-Bafaluy M.P., Acín-Pérez R., Mullikin J.C., Park J.S., Moreno-Loshuertos R., Hu P., Pérez-Martos A., Fernández-Silva P., Bai Y., Enríquez J.A. (2003). Revisiting the Mouse Mitochondrial DNA Sequence. Nucleic Acids Res..

[31] Ferris S.D., Sage R.D., Prager E.M., Ritte U., Wilson A.C. (1983). Mitochondrial DNA Evolution in Mice. Genetics.

[32] Goios A., Pereira L., Bogue M., Macaulay V., Amorim A. (2007). MtDNA Phylogeny and Evolution of Laboratory Mouse Strains. Genome Res..

[33] Yu X., Gimsa U., Wester-Rosenlöf L., Kanitz E., Otten W., Kunz M., Ibrahim S.M. (2009). Dissecting the Effects of MtDNA Variations on Complex Traits Using Mouse Conplastic Strains. Genome Res..

[34] Sitaru C., Mihai S., Otto C., Chiriac M.T., Hausser I., Dotterweich B., Saito H., Rose C., Ishiko A., Zillikens D. (2005). Induction of Dermal-Epidermal Separation in Mice by Passive Transfer of Antibodies Specific to Type VII Collagen. J. Clin. Investig..

[35] van der Fits L., Mourits S., Voerman J.S.A., Kant M., Boon L., Laman J.D., Cornelissen F., Mus A.-M., Florencia E., Prens E.P. (2009). Imiquimod-Induced Psoriasis-like Skin Inflammation in Mice Is Mediated via the IL-23/IL-17 Axis. J. Immunol..

[36] Schröder T., Kucharczyk D., Bär F., Pagel R., Derer S., Jendrek S.T., Sünderhauf A., Brethack A.-K., Hirose M., Möller S. (2016). Mitochondrial Gene Polymorphisms Alter Hepatic Cellular Energy Metabolism and Aggravate Diet-Induced Non-Alcoholic Steatohepatitis. Mol. Metab..

[37] Duscha A., Gisevius B., Hirschberg S., Yissachar N., Stangl G.I., Eilers E., Bader V., Haase S., Kaisler J., David C. (2020). Propionic Acid Shapes the Multiple Sclerosis Disease Course by an Immunomodulatory Mechanism. Cell.

[38] Haghikia A., Jörg S., Duscha A., Berg J., Manzel A., Waschbisch A., Hammer A., Lee D.-H., May C., Wilck N. (2015). Dietary Fatty Acids Directly Impact Central Nervous System Autoimmunity via the Small Intestine. Immunity.

[39] Wallace D.C. (2018). Mitochondrial Genetic Medicine. Nat. Genet..

[40] Shoffner J.M., Lott M.T., Lezza A.M., Seibel P., Ballinger S.W., Wallace D.C. (1990). Myoclonic Epilepsy and Ragged-Red Fiber Disease (MERRF) Is Associated with a Mitochondrial DNA TRNA(Lys) Mutation. Cell.

[41] Buck M.D., O’Sullivan D., Pearce E.L. (2015). T Cell Metabolism Drives Immunity. J. Exp. Med..

[42] Nakahira K., Haspel J.A., Rathinam V.A.K., Lee S.-J., Dolinay T., Lam H.C., Englert J.A., Rabinovitch M., Cernadas M., Kim H.P. (2011). Autophagy Proteins Regulate Innate Immune Responses by Inhibiting the Release of Mitochondrial DNA Mediated by the NALP3 Inflammasome. Nat. Immunol..

[43] Hu X., Wang Y., Hao L.-Y., Liu X., Lesch C., Sanchez B.M., Wendling J.M., Morgan R.W., Aicher T.D., Carter L.L. (2015). Sterol Metabolism Controls TH17 Differentiation by Generating Endogenous RORγ Agonists. Nat. Chem. Biol..

[44] Tian X., Hellman J., Horswill A.R., Crosby H.A., Francis K.P., Prakash A. (2019). Elevated Gut Microbiome-Derived Propionate Levels Are Associated With Reduced Sterile Lung Inflammation and Bacterial Immunity in Mice. Front. Microbiol..

[45] Hirose M., Künstner A., Schilf P., Sünderhauf A., Rupp J., Jöhren O., Schwaninger M., Sina C., Baines J.F., Ibrahim S.M. (2017). Mitochondrial Gene Polymorphism Is Associated with Gut Microbial Communities in Mice. Sci. Rep..

[46] Reichardt N., Duncan S.H., Young P., Belenguer A., McWilliam Leitch C., Scott K.P., Flint H.J., Louis P. (2014). Phylogenetic Distribution of Three Pathways for Propionate Production within the Human Gut Microbiota. ISME J..

[47] Siliprandi N., Di Lisa F., Menabò R. (1991). Propionyl-L-Carnitine: Biochemical Significance and Possible Role in Cardiac Metabolism. Cardiovasc. Drugs Ther..

[48] Chakievska L., Holtsche M.M., Künstner A., Goletz S., Petersen B.-S., Thaci D., Ibrahim S.M., Ludwig R.J., Franke A., Sadik C.D. (2019). IL-17A Is Functionally Relevant and a Potential Therapeutic Target in Bullous Pemphigoid. J. Autoimmun..

[49] Boutet M.-A., Nerviani A., Gallo Afflitto G., Pitzalis C. (2018). Role of the IL-23/IL-17 Axis in Psoriasis and Psoriatic Arthritis: The Clinical Importance of Its Divergence in Skin and Joints. Int. J. Mol. Sci..

[50] Corrêa-Oliveira R., Fachi J.L., Vieira A., Sato F.T., Vinolo M.A.R. (2016). Regulation of Immune Cell Function by Short-Chain Fatty Acids. Clin. Transl. Immunol..

[51] Perry R.J., Borders C.B., Cline G.W., Zhang X.M., Alves T.C., Petersen K.F., Rothman D.L., Kibbey R.G., Shulman G.I. (2016). Propionate Increases Hepatic Pyruvate Cycling and Anaplerosis and Alters Mitochondrial Metabolism. J. Biol. Chem..

[52] Aoyama M., Kotani J., Usami M. (2010). Butyrate and Propionate Induced Activated or Non-Activated Neutrophil Apoptosis via HDAC Inhibitor Activity but without Activating GPR-41/GPR-43 Pathways. Nutrition.

[53] Trompette A., Gollwitzer E.S., Yadava K., Sichelstiel A.K., Sprenger N., Ngom-Bru C., Blanchard C., Junt T., Nicod L.P., Harris N.L. (2014). Gut Microbiota Metabolism of Dietary Fiber Influences Allergic Airway Disease and Hematopoiesis. Nat. Med..

[54] Boominathan A., Vanhoozer S., Basisty N., Powers K., Crampton A.L., Wang X., Friedricks N., Schilling B., Brand M.D., O’Connor M.S. (2016). Stable Nuclear Expression of ATP8 and ATP6 Genes Rescues a MtDNA Complex V Null Mutant. Nucleic Acids Res..

[55] Bornhövd C., Vogel F., Neupert W., Reichert A.S. (2006). Mitochondrial Membrane Potential Is Dependent on the Oligomeric State of F1F0-ATP Synthase Supracomplexes. J. Biol. Chem..

[56] Davies K.M., Anselmi C., Wittig I., Faraldo-Gomez J.D., Kuhlbrandt W. (2012). Structure of the Yeast F1Fo-ATP Synthase Dimer and Its Role in Shaping the Mitochondrial Cristae. Proc. Natl. Acad. Sci. USA.

[57] Wittig I., Meyer B., Heide H., Steger M., Bleier L., Wumaier Z., Karas M., Schägger H. (2010). Assembly and Oligomerization of Human ATP Synthase Lacking Mitochondrial Subunits a and A6L. Biochim. Biophys. Acta Bioenerg..

[58] Allaman I., Bélanger M., Magistretti P.J. (2015). Methylglyoxal, the Dark Side of Glycolysis. Front. Neurosci..

[59] Nokin M.-J., Durieux F., Bellier J., Peulen O., Uchida K., Spiegel D.A., Cochrane J.R., Hutton C.A., Castronovo V., Bellahcène A. (2017). Hormetic Potential of Methylglyoxal, a Side-Product of Glycolysis, in Switching Tumours from Growth to Death. Sci. Rep..

[60] Scharping N.E., Menk A.V., Moreci R.S., Whetstone R.D., Dadey R.E., Watkins S.C., Ferris R.L., Delgoffe G.M. (2016). The Tumor Microenvironment Represses T Cell Mitochondrial Biogenesis to Drive Intratumoral T Cell Metabolic Insufficiency and Dysfunction. Immunity.

[61] Yin Y., Choi S.-C., Xu Z., Perry D.J., Seay H., Croker B.P., Sobel E.S., Brusko T.M., Morel L. (2015). Normalization of CD4+ T Cell Metabolism Reverses Lupus. Sci. Transl. Med..

[62] Quiros P.M., Goyal A., Jha P., Auwerx J. (2017). Analysis of MtDNA/NDNA Ratio in Mice. Curr. Protoc. Mouse Biol..

[63] Chacko B.K., Kramer P.A., Ravi S., Johnson M.S., Hardy R.W., Ballinger S.W., Darley-Usmar V.M. (2013). Methods for Defining Distinct Bioenergetic Profiles in Platelets, Lymphocytes, Monocytes, and Neutrophils, and the Oxidative Burst from Human Blood. Lab. Investig..

[64] Kramer P.A., Ravi S., Chacko B., Johnson M.S., Darley-Usmar V.M. (2014). A Review of the Mitochondrial and Glycolytic Metabolism in Human Platelets and Leukocytes: Implications for Their Use as Bioenergetic Biomarkers. Redox Biol..

[65] Rogers G.W., Brand M.D., Petrosyan S., Ashok D., Elorza A.A., Ferrick D.A., Murphy A.N. (2011). High Throughput Microplate Respiratory Measurements Using Minimal Quantities of Isolated Mitochondria. PLoS ONE.

[66] Iwata H., Witte M., Samavedam U.K.S.R.L., Gupta Y., Shimizu A., Ishiko A., Schroder T., Seeger K., Dahlke M., Rades D. (2015). Radiosensitive Hematopoietic Cells Determine the Extent of Skin Inflammation in Experimental Epidermolysis Bullosa Acquisita. J. Immunol..

[67] Evsikov A.V., Dolan M.E., Genrich M.P., Patek E., Bult C.J. (2009). MouseCyc: A Curated Biochemical Pathways Database for the Laboratory Mouse. Genome Biol..

[68] Caspi R., Billington R., Ferrer L., Foerster H., Fulcher C.A., Keseler I.M., Kothari A., Krummenacker M., Latendresse M., Mueller L.A. (2016). The MetaCyc Database of Metabolic Pathways and Enzymes and the BioCyc Collection of Pathway/Genome Databases. Nucleic Acids Res..

[69] Csardi G., Nepusz T. (2006). The Igraph Software Package for Complex Network Research. Complex Syst..

[70] Sezin T., Zillikens D., Sadik C. (2015). Leukotrienes Do Not Modulate the Course of Aldara^TM^-Induced Psoriasiform Dermatitis in Mice. Acta Derm. Venereol..

