# Supplementary material for: A Mitochondrial Polymorphism Alters Immune Cell Metabolism and Protects Mice from Skin Inflammation
Source: Int J Mol Sci. 2021 Jan 20;22(3):1006. doi: 10.3390/ijms22031006 (PMC7863969; doi:10.3390/ijms22031006)
Supplement: Supplementary file 1 [file ijms-22-01006-s001.zip › ijms-1073631-supplementary_proof.pdf]

Supplementary materials

Figure S1. Data related to Figure 1.

Figure S2. Data related to Figure 2.

Figure S3. Data related to Figure 3.

Figure S4. Differential clinical presentation of B6-mt^{FVB} and B6 mice in Imiquimod (IMQ)-induced skin inflammation model.

Table S1. List of mitochondrial genome variations in B6-mt^{FVB} and B6 mice.

Table S2. List of potential metabolites differentially expressed in liver samples from B6-mt^{FVB} and B6 mice based on untargeted metabolomics.

Table S3. Nuclear genome difference between B6-mt^{FVB} and B6 mice.

Data S1. Networks identified in an integrative network analysis of metabolomics and gene array data sets.

Supplementary figures

Figure S1

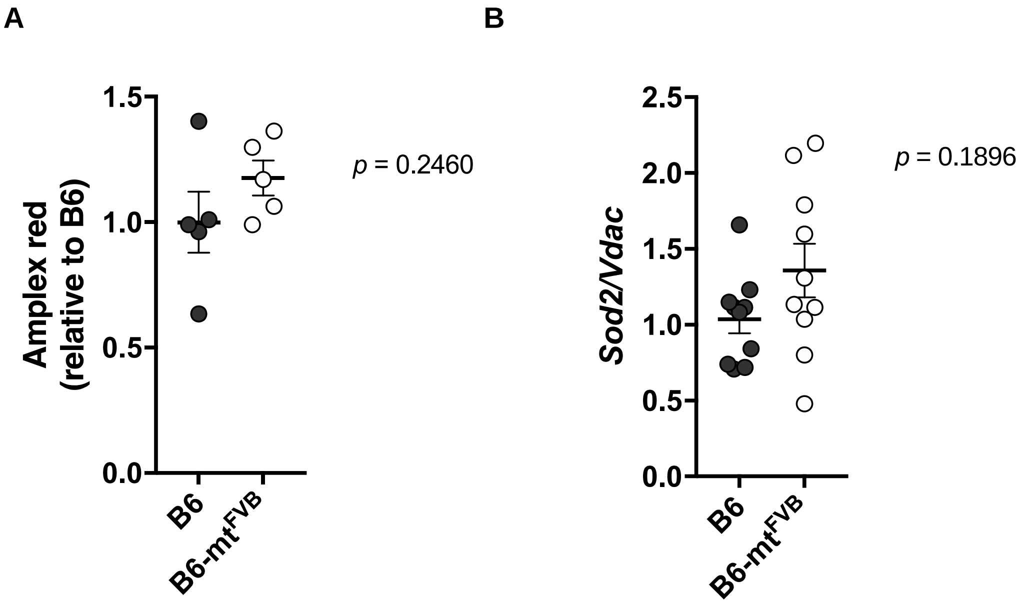

Figure S1. Data related to Figure 1.

A. Hydrogen peroxide levels in liver mitochondria were determined after 30 min of reaction in the assay medium glutamate, malate. No difference was observed between B6-mt^{FVB} and B6 mice. p = 0.2460, Mann-Whitney *U* test. N = 5 / strain, female, 2 months old.

B. The gene expression levels of *Sod2* gene in mitochondrial liver samples were determined by qPCR. B6-mt^{FVB} mice showed a trend of greater *Sod2* expression levels than B6 mice. p = 0.1896, Mann-Whitney *U* test, n = 10 / strain, female, 3 to 4 months old.

Figure S2

A

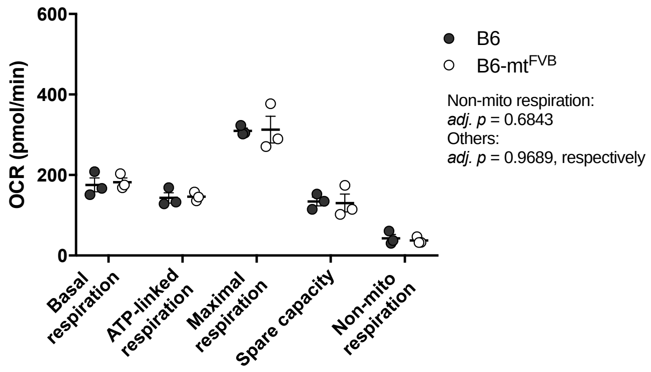

B

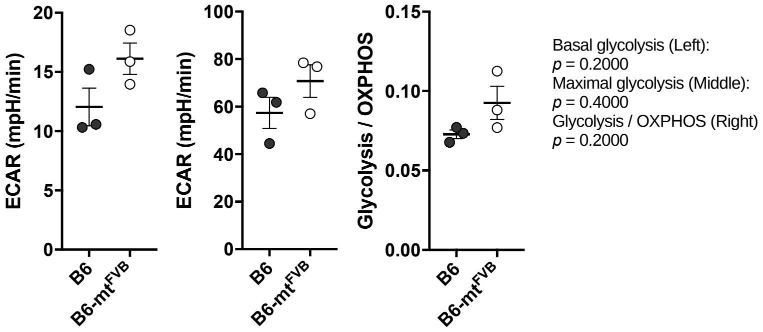

C

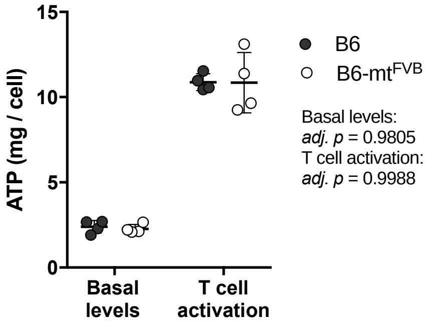

D

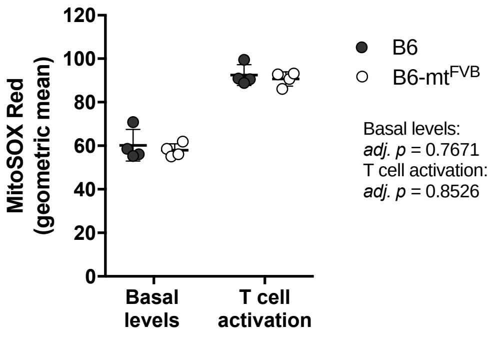

E

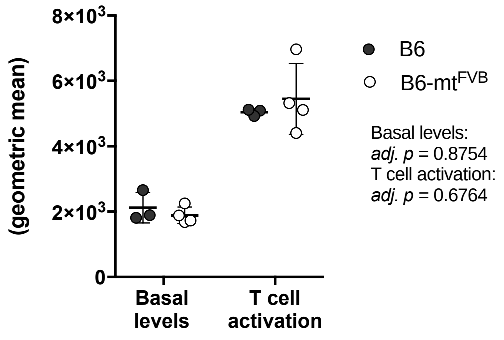

F

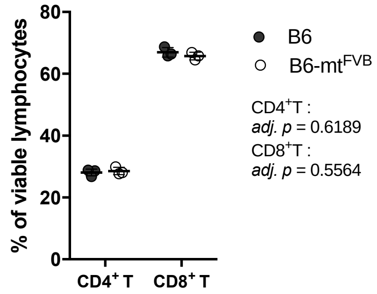

G

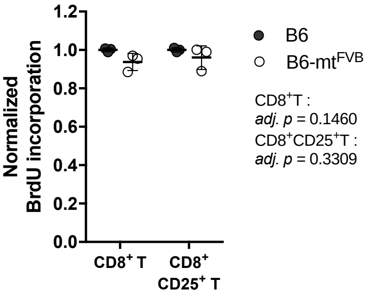

H

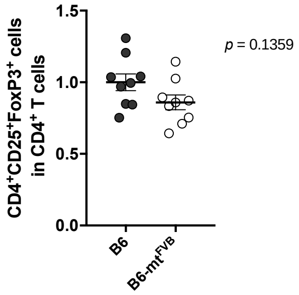

I

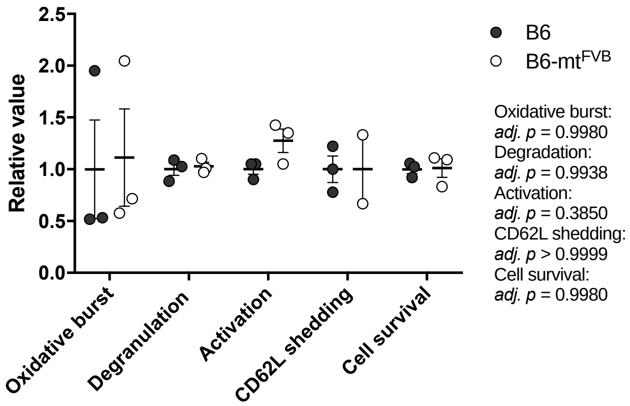

Figure S2. Data related to Figure 2.

A. Mitochondrial oxygen consumption rate in B cells isolated from B6-mt^{FVB} and B6 mice were accessed using Seahorse XF bioanalyzer. Basal respiration, ATP-linked respiration, maximal respiration, spare capacity and non-mitochondrial respiration levels were calculated, and compared between the strains. No significant differences in these values were detected between the strains. *Adj.* p = 0.9689 (basal respiration, ATP-linked respiration, maximal respiration and spare capacity), *adj.* p = 0.6846 (non-mitochondrial respiration), multiple *t* test. N = 3 / strain, female, 3 to 4 months old.

B. Extra cellular acidification rate (ECAR) was determined in the same experiments of (b). Left: Basal glycolysis levels were calculated, and the levels in B cells isolated from B6-mt^{FVB} exhibited higher (16.13 ± 1.328) than those in cells from B6 mice (12.05 ± 1.593). p = 0.2000, Mann-Whitney U test. Middle: Maximal glycolysis levels were higher in B cells from B6-mt^{FVB} (48.34 ± 5.074) than those from B6 mice (38.18 ± 4.402). p = 0.4000, Mann-Whitney U test. Right: A ratio of basal glycolysis levels to basal respiration levels in B cells was calculated, and that of B6-mt^{FVB} mice (0.09259 ± 0.0105) exhibited higher ratio than that of B6 (0.0728 ± 0.0002752). p = 0.2000, Mann-Whitney *U* test.

C. Levels of ATP in CD4$^+$ T cells isolated from B6-mt^{FVB} and B6 were determined immediately after the cells were isolated (basal levels) and after the cells were stimulated with anti-CD3 and anti-CD28 antibodies (T cell activation) for 24 hours. No significant difference in ATP levels in CD4$^+$T cells was detected at both conditions. Basal levels, *adj. p* = 0.9805; activation, *adj. p* = 0.9988; two-way ANOVA. N = 4 (3 males and 1 female) / strain, 3 to 4 months old.

D. Mitochondrial superoxide levels in CD4$^+$ T cells isolated from B6-mt^{FVB} and B6 mice were measured using MitoSOX^{TM} by flowcytometry. The CD4+ T cells were prepared from the same mice used in the experiment (A). CD4$^+$ T cells were activated with anti-CD3 and anti-CD28 antibodies for 24 hours. The levels of MitoSOX in CD4+ T cells were unaltered between the strains at basal levels (*adj. p* = 0.7671) and after T cell activation (*adj. p* = 0.8526). Two-way ANOVA.

E. Levels of mitochondrial membrane potential in CD4$^+$ T cells were comparable between cells from B6-mt^{FVB} and those from B6 mice at basal levels (*adj. p* = 0.8754) and after T cell activation (*adj. p* = 0.6764). Two-way ANOVA. CD4$^+$ T cells used in this experiment were prepared and treated the same as cells in experiment (D). TMRE, tetramethylrhodamine, ethyl ester.

F. The percentage of CD4$^+$ T cells and CD8$^+$ T cells in T cell-activated lymphocytes with anti-mouse CD3 and anti-mouse CD28 antibodies was comparable between B6-mt^{FVB} and B6 mice. The same cells analysed in **Figure 2E**. CD4$^+$ T cells, *adj. p* = 0.6189; CD8$^+$ T cells, *adj. p* = 0.5564; multiple *t* test. N = 3 / strain, males, 3 months of age.

G. Cell proliferation upon the activation with anti-mouse CD3 and anti-mouse CD28 antibodies was determined in CD8$^+$ T cells isolated from B6-mt^{FVB} and B6 mice. Incorporated BrdU levels normalized to the average of those in cells from B6 mice are displayed. BrdU levels in CD8$^+$ T cells and CD8$^+$ CD25$^+$ T cells from B6-mt^{FVB} mice were comparable to those from B6 mice. *adj. p* = 0.1460, *adj. p* = 0.3309, respectively; multiple *t* test. The data is from the same cells analysed in **Figure 2E**.

H. The proportion of CD4$^+$ CD25$^+$ FoxP3$^+$ T cells (i.e., regulatory T cells) was comparable between B6-mt^{FVB} mice and B6 mice. *p* = 0.1359, Mann-Whitney *U* test, n = 9 / strain, males, 3 months of age.

I. A panel of neutrophils functions were assessed in bone marrow - derived neutrophils isolated from B6-mt^{FVB} and B6 mice. Levels of oxidative burst (DHR123 signal intensity), degranulation (CD11b signal intensity), activation (CD69 signal intensity), CD62L shedding (CD62L positive population), and cell survival (annexin V / PI negative population) were compared and all values were unaltered between the neutrophils from two strains. *Adj. p* = 0.9980, *adj. p* = 0.9938, *adj. p* = 0.3850, *adj. p* > 0.9999, and *adj. p* = 0.9980, respectively; multiple *t* test. N = 3 / strain.

Figure S3

A

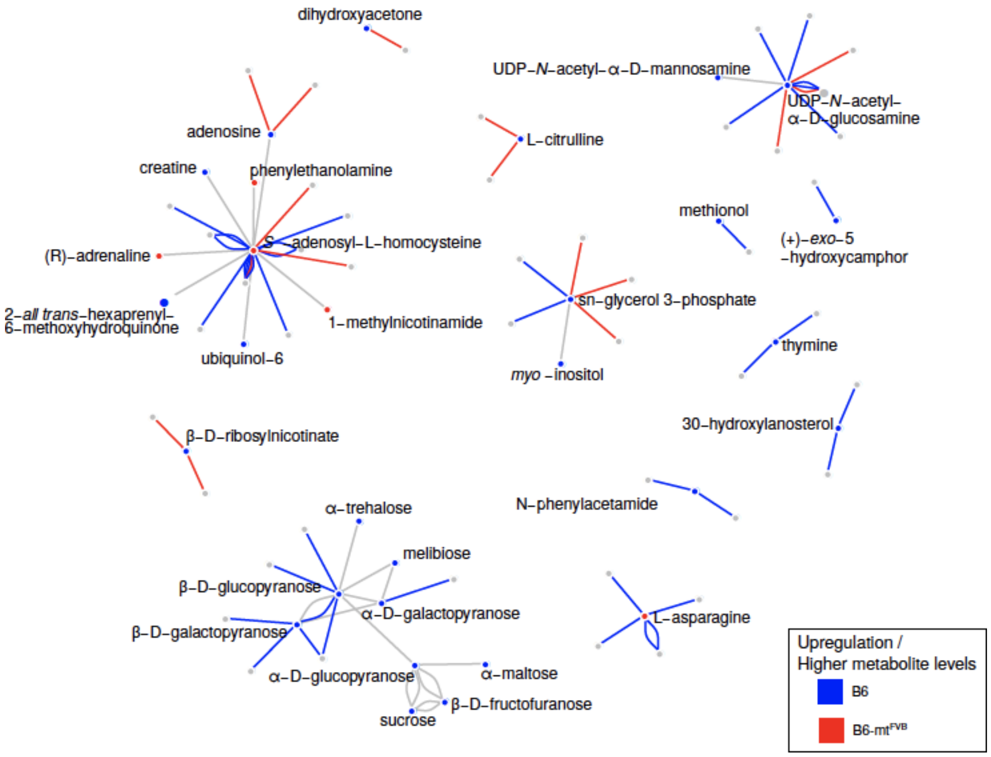

B

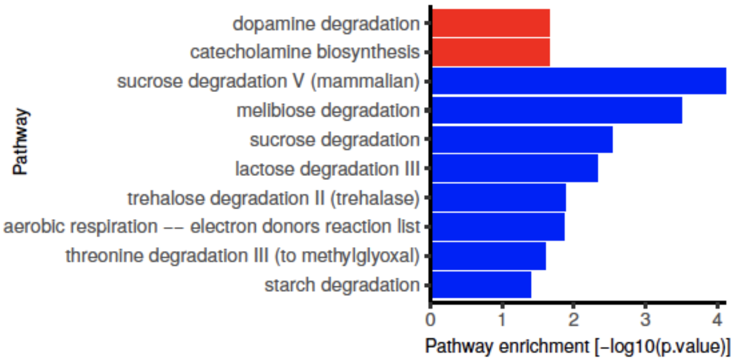

Figure S3. Data related to Figure 3.

A. The network of metabolites identified in an integrative analysis of the untargeted metabolomics data and the gene array data. Vertices represent metabolites, and edges represent reactions where two metabolites are connected if the metabolites participate at least in one common metabolic reaction. Nodes and edges are coloured (red or blue) if the metabolite corresponds by its mass to one of the significant features in the metabolomics data as listed in **Table S2**. Edges are coloured if at least one gene, that is associated with the respective metabolic reaction (i.e., enzyme-coding gene), is differentially expressed (red, up-regulated; blue, down-regulated) in B6-mt^{FVB} mice compared to B6 mice.

B. Results from pathway-enrichment-analysis of metabolic pathways from the MouseCyc-Database and metabolites and metabolites that were predicted to be significantly abundant (see **Supplementary material data S1**) in either B6 (blue) or B6-mt^{FVB} mice (red) based on the untargeted metabolomics data.

Figure S4

A

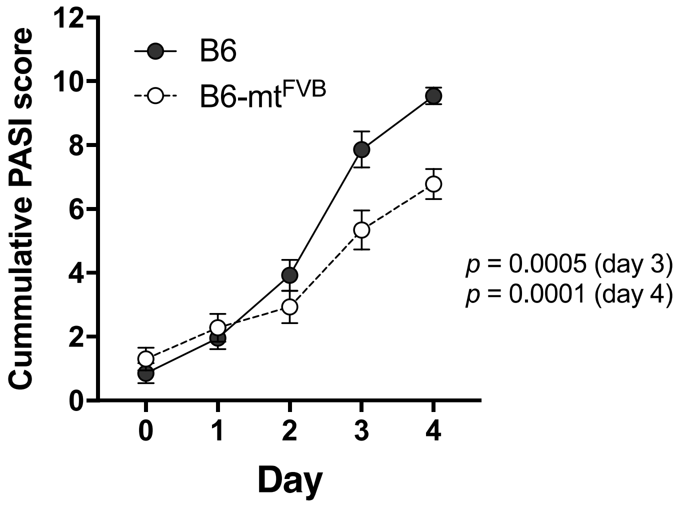

B

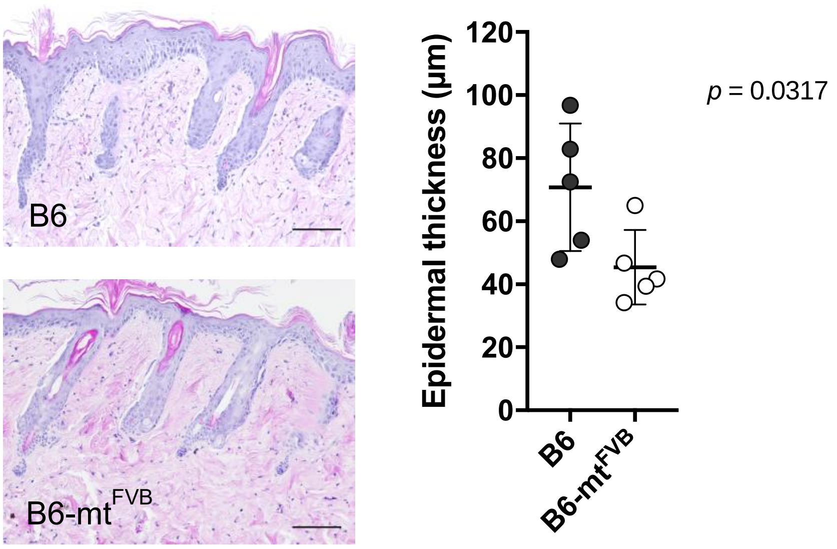

Figure S4. Differential clinical presentation of B6-mt^{FVB} and B6 mice in Imiquimod (IMQ)-induced skin inflammation model.

A. Disease severity of IMQ-treated mice was evaluated using psoriasis activity score index (PASI). B6-mt^{FVB} mice demonstrated significantly less PASI score compared to B6 mice at day 3 (p = 0.0005) and day 4 (p = 0.0001). Two-way ANOVA. B6, n = 23 (14 males and 9 females); B6-mt^{FVB}, n = 14 (12 males and 4 females).

B. Left: Representative picture of histopathology of the back skin in the experiment (A). Bar indicates 100 µm. Original picture magnification is ×200. Right: The epidermis thickness was measured at 8 different spots of a section under the magnification of ×100. p = 0.0317, Mann-Whitney U test.

Supplementary tables

Table S1. Table of mtDNA variations in B6-mt^{FVB} and B6 mice.

Position in mtDNA	7778	9461	9821
Strain | Gene	*mt-Atp8*	*mt-Nd3*	*mt-Tr*
B6-mt^{FVB}	T	C	9A
B6	G	T	8A
AA change	Asp - Tyr	Met - Met	-

Table S2. List of potential metabolites differentially expressed in liver samples from B6-mt^{FVB} and B6 mice based on untargeted metabolomics.

Compound	Formula	P-value	Ionisation mode	Adduct	m/z	LCMS column*
Higher levels in B6-mt^{FVB}						
***S*-adenosyl-L-homocysteine**	C14H20N6O5S	0.0029	positive	M+H+Na	204.0590	RP
***trans*-zeatin-*O*-glucoside-7-*N*-glucoside**	C22H33N5O11	0.0029	positive	M+3Na	204.0618	RP
**indole-3-glyoxal**	C10H7NO2	0.0045	positive	M+CH3OH+H	206.0812	RP
**phenylethanolamine**	C8H11NO	0.0045	positive	M+H+HCOONa	206.0788	RP
***N,N*-dimethylaniline-*N*-oxide**	C8H11NO	0.0045	positive	M+H+HCOONa	206.0788	RP
**L-epinephrine**	C9H13NO3	0.0045	positive	M+Na	206.0788	RP
**enol-phenylpyruvate**	C9H8O3	0.0045	positive	M+ACN+H	206.0812	RP
**3-phenyl-2-oxopropanoate**	C9H8O3	0.0045	positive	M+ACN+H	206.0812	RP
**L-tyrosine methyl ester 4-sulfate**	C10H13NO6S	0.0284	negative	M+FA-H	319.0367	RP
***O*-octanoyl-L-carnitine**	C15H29NO4	0.0284	positive	M+H+HCOONa	356.2044	RP
**coenzyme A**	C21H36N7O16P3S	0.0284	negative	M+ACN-H	807.1345	RP
**1-methylnicotinamide**	C7H8N2O	0.0332	negative	2M+Hac-H	331.1412	RP
**thiamine**	C12H16N4OS	0.0387	positive	M+Na	287.0937	RP
**uroporphyrinogen-III**	C40H44N4O16	0.0387	positive	M+2H+Na	287.0930	RP
**L-asparagine**	C4H8N2O3	0.0387	positive	2M+Na	287.0962	RP
**3-ureidopropanoate**	C4H8N2O3	0.0387	positive	2M+Na	287.0962	RP
**3,4-dihydroxyphenylacetaldehyde**	C8H8O3	0.0387	positive	2M+H-H2O	287.0914	RP
**(R,R)-2,3-butanediol**	C4H10O2	0.0449	positive	M+2Na-H	135.0392	RP
**(R,S)-2,3-butanediol**	C4H10O2	0.0449	positive	M+2Na-H	135.0392	RP
**(S,S)-2,3-butanediol**	C4H10O2	0.0449	positive	M+2Na-H	135.0392	RP
***N*2-formyl-*N*1-(5-phospho-beta-D-ribosyl)glycinamide**	C8H15N2O9P	0.0449	positive	M+H+HCOONa	383.0462	RP
**indole-5,6-quinone-2-carboxylate**	C9H5NO4	0.0449	positive	2M+H	383.0510	RP

Higher levels in B6						
**adenosine**	C10H13N5O4	0.0056	negative	M+Br	346.0156	RP
**2'-deoxyguanosine**	C10H13N5O4	0.0056	negative	M+Br	346.0156	RP
***cis*-aconitate**	C6H6O6	0.0056	negative	2M+FA-H	392.0233	RP
**L-dehydro-ascorbate**	C6H6O6	0.0056	negative	2M+FA-H	392.0233	RP
**4-imidazoleacetate**	C5H6N2O2	0.0083	positive	M+NH4-H2O	126.0662	RP
**thymine**	C5H6N2O2	0.0083	positive	M+NH4-H2O	126.0662	RP
**cotinine methonium ion**	C11H14N2O	0.0121	positive	M+ACN+Na	254.1264	RP
***O*-octanoyl-L-carnitine**	C15H29NO4	0.0121	positive	M+H+2K	122.0478	RP
**methionol**	C4H10OS	0.0121	positive	2M+ACN+H	254.1243	RP
**creatine**	C4H9N3O2	0.0121	negative	M+Hac-H	190.0833	RP
**adenosine**	C10H13N5O4	0.0056	negative	M+Br	346.0156	RP
**2'-deoxyguanosine**	C10H13N5O4	0.0056	negative	M+Br	346.0156	RP
***cis*-aconitate**	C6H6O6	0.0056	negative	2M+FA-H	392.0233	RP
**L-dehydro-ascorbate**	C6H6O6	0.0056	negative	2M+FA-H	392.0233	RP
**4-imidazoleacetate**	C5H6N2O2	0.0083	positive	M+NH4-H2O	126.0662	HILIC
**thymine**	C5H6N2O2	0.0083	positive	M+NH4-H2O	126.0662	HILIC
**cotinine methonium ion**	C11H14N2O	0.0121	positive	M+ACN+Na	254.1264	RP
***O*-octanoyl-L-carnitine**	C15H29NO4	0.0121	positive	M+H+2K	122.0478	RP
**methionol**	C4H10OS	0.0121	positive	2M+ACN+H	254.1243	RP
**creatine**	C4H9N3O2	0.0121	negative	M+Hac-H	190.0833	RP
**3-dimethylsulfoniopropionaldehyde**	C5H10OS	0.0121	positive	2M+NH4	254.1243	RP
**L-citrulline**	C6H13N3O3	0.0121	negative	M-H+O	190.0833	RP
**acetate**	C2H4O2	0.0205	negative	3M-H	179.0561	RP
**(*S*)-lactate**	C3H6O3	0.0205	negative	2M-H	179.0561	RP
**3-hydroxypropanoate**	C3H6O3	0.0205	negative	2M-H	179.0561	RP
**D-glyceraldehyde**	C3H6O3	0.0205	negative	2M-H	179.0561	RP
**(*R*)-lactate**	C3H6O3	0.0205	negative	2M-H	179.0561	RP

**dihydroxyacetone**	C3H6O3	0.0205	negative	2M-H	179.0561	RP
**alpha-D-glucopyranose**	C6H12O6	0.0205	negative	M-H	179.0561	RP
**beta-D-glucopyranose**	C6H12O6	0.0205	negative	M-H	179.0561	RP
**beta-D-fructofuranose**	C6H12O6	0.0205	negative	M-H	179.0561	RP
**alpha-D-galactopyranose**	C6H12O6	0.0205	negative	M-H	179.0561	RP
**beta-D-galactopyranose**	C6H12O6	0.0205	negative	M-H	179.0561	RP
***myo*-inositol**	C6H12O6	0.0205	negative	M-H	179.0561	RP
***N*-phenylacetamide**	C8H9NO	0.0205	negative	M+FA-H	179.0588	RP
**geranate**	C10H16O2	0.0242	positive	2M+ACN+Na	400.2458	RP
**(+)-*exo*-5-hydroxycamphor**	C10H16O2	0.0242	positive	2M+ACN+Na	400.2458	RP
**(5*S*)-HPETE**	C20H32O4	0.0242	positive	M+ACN+Na	400.2458	RP
**(12*S*)-HPETE**	C20H32O4	0.0242	positive	M+ACN+Na	400.2458	RP
**(15*S*)-HPETE**	C20H32O4	0.0242	positive	M+ACN+Na	400.2458	RP
**leukotriene B4**	C20H32O4	0.0242	positive	M+ACN+Na	400.2458	RP
**30-hydroxylanosterol**	C30H50O2	0.0242	positive	M+2Na-H	487.3522	HILIC
**4,4-dimethyl-14alpha-formyl-5alpha-cholesta-8-en-3beta-ol**	C30H50O2	0.0242	positive	M+2Na-H	487.3522	HILIC
**ubiquinol-6**	C39H60O4	0.0242	positive	M+CH3OH+H	625.4827	HILIC
**beta-D-ribosylnicotinate**	C11H13NO6	0.0284	negative	M-H+HCOONa	322.0544	RP
**(*S*)-5-hydroxyisourate**	C5H4N4O4	0.0284	positive	M+ACN+H	226.0571	RP
**L-saccharopine**	C11H20N2O6	0.0332	positive	2M+2H+3H2O	304.1553	RP
**UDP-*N*-acetyl-alpha-D-glucosamine**	C17H27N3O17P2	0.0332	positive	M+Li	614.0971	RP
**UDP-*N*-acetyl-alpha-D-mannosamine**	C17H27N3O17P2	0.0332	positive	M+Li	614.0971	RP
**trehalose**	C12H22O11	0.0387	positive	M+ACN+H	384.1500	RP
**alpha-maltose**	C12H22O11	0.0387	positive	M+ACN+H	384.1500	RP
**melibiose**	C12H22O11	0.0387	positive	M+ACN+H	384.1500	RP
**sucrose**	C12H22O11	0.0387	positive	M+ACN+H	384.1500	RP
**beta-D-galactosyl-(1,4)-*N*-acetyl-D-glucosamine**	C14H25NO11	0.0387	positive	M+H	384.1500	RP

**nicotinate adenine dinucleotide**	C21H26N6O15P2	0.0387	positive	M-NH3+H	648.0739	RP
***sn*-glycerol 3-phosphate**	C3H9O6P	0.0387	negative	M+Hac-H	231.0275	RP
**D-gluconate**	C6H12O7	0.0387	negative	M+Cl	231.0277	RP
**L-gulonate**	C6H12O7	0.0387	negative	M+Cl	231.0277	RP
**enol-phenylpyruvate**	C9H8O3	0.0387	negative	M-H+HCOONa	231.0275	RP
**3-phenyl-2-oxopropanoate**	C9H8O3	0.0387	negative	M-H+HCOONa	231.0275	RP
**aminoacetone**	C3H7NO	0.0449	positive	M+H+HCOOK	158.0214	RP
**3-aminopropanal**	C3H7NO	0.0449	positive	M+H+HCOOK	158.0214	RP
***O*-phospho-L-serine**	C3H8NO6P	0.0449	positive	M-CO+H	158.0213	RP
**L-threonine**	C4H9NO3	0.0449	positive	M+K	158.0214	RP
**D-threonine**	C4H9NO3	0.0449	positive	M+K	158.0214	RP
**5-guanidino-2-oxopentanoate**	C6H11N3O3	0.0449	negative	M+Hac-H	232.0939	RP
**2-*alltrans*-hexaprenyl-6-methoxyhydroquinone**	C37H56O3	0.0449	positive	M+Li	555.4385	HILIC

*RP, reverse-phase; HILIC, hydrophilic interaction chromatography

Table S3. Nuclear genome difference between B6-mt^{FVB} and B6 mice.

Strain			B6			B6-mt^{FVB}		
Sex			M	F	F	F	F	F
ChrB37	PosB37	SNP	Mouse 1	Mouse 2	Mouse 3	Mouse 1	Mouse 2	Mouse 3
10	80972452	UNC100214691	CC	CC	CC	CA	CC	CA
nDNA homology to B6 (%)			100	100	100	99.999	100	99.999
